# Hybrid SMART spheroids to enhance stem cell therapy for CNS injuries

**DOI:** 10.1126/sciadv.abj2281

**Published:** 2021-09-29

**Authors:** Christopher Rathnam, Letao Yang, Sofia Castro-Pedrido, Jeffrey Luo, Li Cai, Ki-Bum Lee

**Affiliations:** 1 Department of Chemistry and Chemical Biology Rutgers, The State University of New Jersey, Piscataway, NJ 08854, USA.; 2 Department of Biomedical Engineering Rutgers, The State University of New Jersey, Piscataway, NJ 08854, USA.

## Abstract

Although stem cell therapy holds enormous potential for treating debilitating injuries and diseases in the central nervous system, low survival and inefficient differentiation have restricted its clinical applications. Recently, 3D cell culture methods, such as stem cell–based spheroids and organoids, have demonstrated advantages by incorporating tissue-mimetic 3D cell-cell interactions. However, a lack of drug and nutrient diffusion, insufficient cell-matrix interactions, and tedious fabrication procedures have compromised their therapeutic effects in vivo. To address these issues, we developed a biodegradable nanomaterial-templated 3D cell assembly method that enables the formation of hybrid stem cell spheroids with deep drug delivery capabilities and homogeneous incorporation of 3D cell-matrix interactions. Hence, high survival rates, controlled differentiation, and functional recovery were demonstrated in a spinal cord injury animal model. Overall, our hybrid stem cell spheroids represent a substantial development of material-facilitated 3D cell culture systems and can pave the way for stem cell–based treatment of CNS injuries.

## Teaser

A hybrid 3D assembly method was developed to advance spheroid culture and enhance stem cell–based treatment of CNS injuries.

## INTRODUCTION

Neurological disorders, especially central nervous system (CNS) injuries and diseases, are often debilitating and difficult to cure, mainly due to the intrinsically limited capacity for neuroregeneration and complex inhibitory microenvironment in the nervous system (*1*–*4*). Hence, there is an urgent need to develop reliable treatments for neurological disorders by using innovative methods to generate functional neural cells (primarily neurons) and reestablish the damaged neural circuitry. Regarding these challenges, stem cell (cell replacement) therapies would be a promising approach, as they have many therapeutic benefits, including the ability to proliferate, differentiate into functional neural cells, and secrete various immunomodulatory factors (*5*–*7*). Hence, several preclinical and clinical studies have demonstrated the huge potential of stem cell–based therapies ex vivo and in vivo for the effective treatment of many human diseases and disorders (*6*, *8*, *9*). Despite their enormous potential, current stem cell–based treatments against CNS injuries and diseases are substantially restricted by the poor survival rate, inefficient integration, loss of neural plasticity, and uncontrollable differentiation of implanted cells, which is attributed mainly to the highly inhibitory and inflammatory microenvironment at disease or injury sites (*3*, *5*, *10*–*12*).

Addressing the above problems, one promising approach is to use scaffold materials to generate favorable microenvironments during stem cell implantation (*13*–*16*). These scaffolds can help generate three-dimensional (3D) stem cell assembly, mitigate local inflammation, and establish favorable cell–extracellular matrix (ECM) interactions, typically mediated through focal adhesion kinase (FAK) signaling, which critically regulates neurogenesis and axon elongation (*17*–*22*). Nevertheless, a few challenges have been reported in these scaffold-based approaches. For example, mechanical mismatches, improper biodegradation rates, and immune reactions from conventional polymer scaffolding materials have limited their broad clinical applications (*16*, *23*–*25*). The recent development of injectable hydrogels partially addressed these challenges. For instance, self-assembling peptide hydrogels can provide immunomodulatory functions and deliver drugs in a highly programmable and biocompatible manner, which make them very interesting candidates for in vivo therapies; however, the incorporation of critical niches such as cell-cell interactions has not been fully realized as of yet, restricting their broad clinical applications (*17*, *26*–*28*). Hence, most scaffold-based approaches remain suboptimal for CNS applications.

**Fig. 1.**
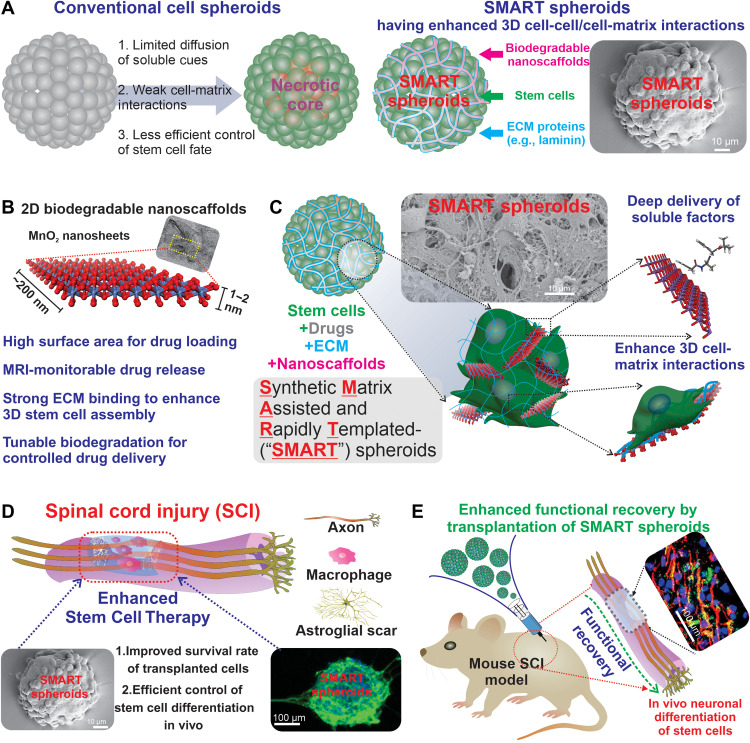
Assembly of SMART spheroids for advanced cell therapy. (**A**) To better mimic the natural structure of 3D tissues and enhance the beneficial properties of stem cells for therapy, we incorporate advanced biodegradable nanomaterials that act as both a bonding agent as well as structural support and a drug delivery vehicle. (**B**) Manganese dioxide nanosheets are the ideal choice for our hybrid system due to their unique physical, chemical, and biological properties. (**C**) 2D nanosheets of manganese dioxide are coated with ECM proteins and drugs and assembled with cells owing to the unique physicochemical properties of the nanomaterials allowing the formation of our SMART spheroids. (**D** and **E**) As a proof of concept, we delivered SMART in an in vivo spinal cord injury (SCI) model. We demonstrated that SMART assembly reliably enhanced the survival of stem cells at both 1-week and 1-month time points while simultaneously increasing the differentiation efficiency of the implanted cells into neurons. This leads to a reduction in glial scar formation and increased levels of neuroprotection, culminating in enhanced motor function recovery.

Another promising strategy to improve stem cell therapies is to generate scaffold-free cell spheroids and their direct injection into the CNS injury sites (*29*–*33*). Stem cell spheroids, which are 3D spherical cellular assemblies, have recently garnered much attention as promising avenues for disease modeling, drug discovery, and stem cell therapy (*34*–*36*). Typically generated from 3D cell culture, spheroid-based scaffold-free stem cell therapies can enhance cell survival and differentiation and stimulate the secretion of neurotrophic factors through their biomimetic 3D cell-cell interactions (*30*, *31*, *37*, *38*). They also allow the injectable delivery of high densities of stem cells at sites of CNS injuries or diseases in an accurate and relatively noninvasive manner, thereby enhancing the therapeutic outcomes of stem cell implantation (*29*, *30*, *32*, *33*). However, several barriers exist in the in vivo translation of spheroid-based stem cell therapy. For example, the controlled differentiation of stem cell spheroids into specific cell lineages (e.g., neurons and glial cells) has mostly relied on the spontaneous cell signaling of stem cells inside neurospheres [spheroids assembled from neural stem cells (NSCs)] (*30*). As a result, there is a lack of cell-ECM interactions and inhomogeneous access of biological cues to the aggregated cells inside the spheroids, often leading to undesired gliogenesis and inefficient neurogenesis. Besides, restricted diffusion of oxygen, nutrients, and growth factors into the core of spheroids can often induce cell apoptosis during long-term culture in vitro, a phenomenon known as “the necrotic core” (*39*). Given the advantages and disadvantages of scaffold- and scaffold-free spheroid-based approaches, it would be desired to develop a combined synergistic approach that can (i) create hybrid stem cell spheroids encompassing 3D cell-cell and cell-matrix interactions in a controlled manner and (ii) enable the controlled, homogeneous, and monitorable drug release inside of spheroids in vivo at disease injury sites, both of which can lead to a breakthrough in stem cell therapies for CNS applications (Fig. 1A).

To this end, we developed a nanobiomaterial-mediated 3D cell assembly method to generate hybrid stem cell spheroids in which controlled cell-matrix interactions and drug release were incorporated. In our proof-of-concept demonstration, biodegradable 2D nanomaterials effectively templated and promoted the rapid assembly of human induced pluripotent stem cell (hiPSC)–derived NSCs (hiPSC-NSCs) into hybrid 3D spheroids. In this way, we can better control the formation of spheroids and their differentiation into functional neurons in vitro and in vivo, resulting in improved therapeutic outcomes in a spinal cord injury (SCI) animal model. To accomplish these goals, manganese dioxide nanosheet was used as an ideal nanomaterial for spheroid formation due to their high drug loading, redox-mediated biodegradation, magnetic resonance imaging (MRI)–active degradation products, and biocompatibility (*20*, *21*, *40*, *41*). Although several other nanomaterials (e.g., graphene nanosheets, gold nanowires, and carbon nanotubes) have been applied for 3D stem cell cultures, most of them are intrinsically nonbiodegradable, do not facilitate the assembly process, and have limited biocompatibility for in vivo applications (*14*, *15*, *18*, *22*, *42*–*45*). In contrast, our advanced 2D nanomaterial-mediated 3D cell assembly method has several advantages over the previous nanomaterial-based approaches. First, we found that the 3D cell assembly process occurs under physiological conditions and is rapidly driven by two key noncovalent interactions: the metal-π/hydrophobic interactions between MnO_2_ nanosheets and ECM proteins (e.g., laminin) and the integrin-binding interactions between ECM proteins and stem cells (Fig. 1B) (*46*). Second, our biodegradable 2D nanomaterial facilitated the formation of 3D stem cell spheroids through effectively enhanced cell-matrix interactions and enabled us to develop a controlled/homogeneous drug release inside the core of spheroids to improve stem cell survival and control their differentiation in vivo. Third, the 2D nanomaterial embedded in the spheroids further led to a unique MRI monitorable drug release, due to a stoichiometric generation of T1 MRI contrast agent (Mn^2+^) and drugs during the biodegradation under redox conditions (Fig. 1C). We named our developed method “synthetic matrix-assisted and rapidly templated” (SMART) assembly, and the hybrid spheroids as “SMART spheroids.” By developing this SMART spheroid technology, we could effectively synergize scaffold-based and scaffold-free approaches to induce in vitro and in vivo neuronal differentiation of stem cell spheroids, thereby paving the way for the potential treatment of CNS injuries such as SCI and traumatic brain injury (Fig. 1, D and E).

## RESULTS AND DISCUSSION

### SMART neurospheres generated by controlled 3D SMART assembly

**Fig. 2.**
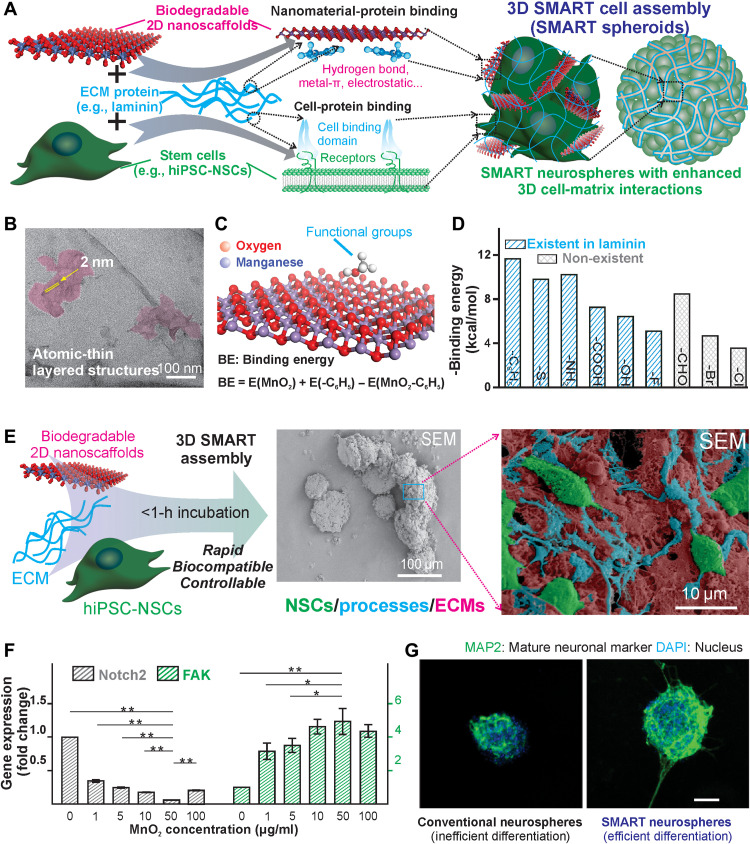
Formation and characterization of the SMART neurospheres. (**A**) The unique bonding of manganese dioxide nanosheets to ECM and drugs through various interactions allows enhanced loading. Subsequent binding of cell-binding domains to the ECM proteins enables a rapid and robust assembly of material interspersed spheroids. (**B**) TEM images of the manganese dioxide nanosheets showing the atomically thin layered structure and size. (**C** and **D**) Simulation using molecular dynamics to study the bonding of common functional groups found in ECM proteins and drugs, which allow the enhanced binding of functional components of SMART neurospheres onto the nanosheets. Reprinted (adapted) with permission from (*46*). Copyright (2018) American Chemical Society. (**E**) The assembly process was visualized using light and electron microscopy, showing the detailed structures of the spheroids generated with nanomaterials embedded in the spheroids as shown by the dark color of the spheroids and the high-magnification SEM. (**F**) The modulation of physical cues, which have been shown to have a notable effect on neuronal differentiation, was characterized using qPCR showing dose-dependent control of *Notch* signaling (cell-cell) and *FAK* signaling (cell-matrix) at culture day 7. Data are means ± SEM; *n* = 4; *P < 0.05, **P < 0.01 by one-way analysis of variance (ANOVA). (**G**) This control of physical cues correlated to an increase in neuronal differentiation as shown by immunostaining of MAP2, a common neuronal marker at culture day 7. Scale bars, 100 μm. DAPI, 4′,6-diamidino-2-phenylindole.

To initiate the formation of a SMART NSC spheroid (termed “SMART neurosphere”), we mixed a solution of NSCs (hiPSC-NSCs, 1 million cells/ml, in neurobasal medium) with a second solution of neural ECM (i.e., laminin protein, 57 kDa)–conjugated nanomaterial (i.e., gamma phase MnO_2_ nanosheets) until cell aggregations occurred in 10 to 15 min (Fig. 2A). Specifically, we selected laminin as a proof-of-concept demonstration, as it is one of the critical neural ECM molecules regulating the survival and neurogenesis of stem cells in the human brain. In addition, hiPSC-NSCs were chosen for their excellent clinical potential for cell therapies (*47*–*49*). MnO_2_ nanosheets served as the last and most crucial building block for the SMART neurospheres by having a thin atomic structure to bind laminin efficiently, a negative surface charge (−25 mV) (fig. S1) to minimize cellular uptake, and a lateral size (~200 nm) (Fig. 2B and fig. S1B) that supports the formation of focal adhesions by encompassing the size of small integrin clusters (*50*). In our SMART assembly process, there are two essential pairs of noncovalent interactions that dominate the formation of the SMART neurospheres: (i) the metal-π/hydrophobic/hydrogen-bonding interactions between MnO_2_ nanosheets and laminin that allowed laminin adsorption on the surface of nanosheets, as simulated by our previous reports showing binding energy of functional groups found in proteins and drugs (Fig. 2, C and D) (*46*), and (ii) the integrin-binding between laminin and NSCs, which allowed 3D spheroid formation. The successful generation of SMART neurospheres was not only demonstrated by the observation of dark-colored aggregations after mixing cells with laminin-conjugated MnO_2_ nanosheets but also characterized by optical microscopy and field-emission scanning electron microscopy (FE-SEM) (Fig. 2E and fig. S2). Specifically, compared to spheroids generated by conventional methods, SMART neurospheres showed hierarchical structures with NSCs interfacing with the nanostructured 3D matrix (Fig. 2E). As a control, incubation of NSCs and laminin alone without MnO_2_ nanosheets under identical conditions did not generate any obvious cell aggregates. Notably, the SMART assembly was nearly an order of magnitude faster than conventional spheroid formation techniques typically based on cell-cell interactions, which is desirable for treating acute conditions of CNS diseases and injuries.

Although the detailed mechanism remains to be investigated, such accelerated spheroid formation could be attributed to higher probabilities of the collision between cells and laminin-coated nanosheets at much higher concentrations (over 5 × 10^8^ fold higher, assuming that the molecular weight of one 50 nm–sized MnO_2_ nanosheets is 3,480,000). Of equal importance, size control [from sub–100 μm (less than 5000 cell aggregates) to above 500 μm (more than 1 million cell aggregates)] of SMART neurospheres, which could fundamentally affect the injectability during implantation and the viability of stem cells at disease/injury sites, was realized by varying concentrations of MnO_2_ nanosheets and further combining a microwell array. However, size-dependent changes in cell behavior and molecular pathways on SMART spheroids remain to be studied (fig. S2). Furthermore, as we incorporated exogenous materials (MnO_2_ nanosheets) into the SMART neurospheres, it was crucial to ensure that the viabilities of the assembled stem cells were not affected. We confirmed the excellent biocompatibility of MnO_2_ nanosheets at our working concentrations of 1 to 50 μg/ml in the SMART neurosphere by a standard PrestoBlue assay (cell viability starts to decrease at 50 μg/ml, probably due to the reduction of cellular bioreductants such as glutathione) (fig. S1). Together, we established and optimized our biocompatible SMART assembly method successfully and generated SMART neurospheres encompassing favorable 3D cell-matrix interfaces.

Furthermore, we hypothesized that incorporating 3D cell-matrix interactions into the SMART neurosphere could better modulate stem cell neurogenesis desired for cell therapies. Although neurospheres hold a great promise for treating CNS injuries and diseases, a lack of cell-matrix interactions remains a critical barrier for the effective induction of neurogenesis. For instance, FAK-associated pathways, typically initiated from cellular interactions with neural ECM molecules such as laminin, play an essential role in the neurogenesis of stem cells. However, such beneficial FAK pathways are often suppressed due to the dominating cell-cell interactions in neurospheres, resulting in less controlled differentiation of stem cells (*51*–*57*). In this regard, we verified that SMART neurospheres, incorporating 3D cell-matrix interactions, induced significantly higher expression of *FAK* compared to control spheroids (Fig. 2F). Besides, such cell-matrix interactions could be modulated effectively by merely varying the concentration (1 to 0 μg/ml MnO_2_ nanosheets) of nanosheets during assembly, as shown by mRNA expression analysis using quantitative real-time polymerase chain reaction (qRT-PCR) (Fig. 2F and table S1). Moreover, the up-regulation of *FAK* in SMART neurospheres further led to reduced cell-cell interactions, as partially supported by qRT-PCR analysis of *Notch* gene expression, although more detailed protein analysis would be required (Fig. 2F). As a result, neurogenesis was significantly improved in our SMART neurosphere-based stem cell differentiation assay with an enhancement of axonal growth by 6.9-fold (Fig. 2G and figs. S3 and S4). In addition, to confirm the important role of FAK signaling in spheroid formation, we treated a FAK inhibitor to cells before spheroid formation. As a result, spheroids could not form densely packed spheroids but rather mostly lacked any assembly of cells or formed loosely assembled aggregates (fig. S5). Through these experiments, we validated that more effective control over spheroid neurogenesis could be achieved by incorporating 3D cell-matrix interactions into SMART neurospheres.

### Investigating deep drug delivery in SMART neurospheres

**Fig. 3.**
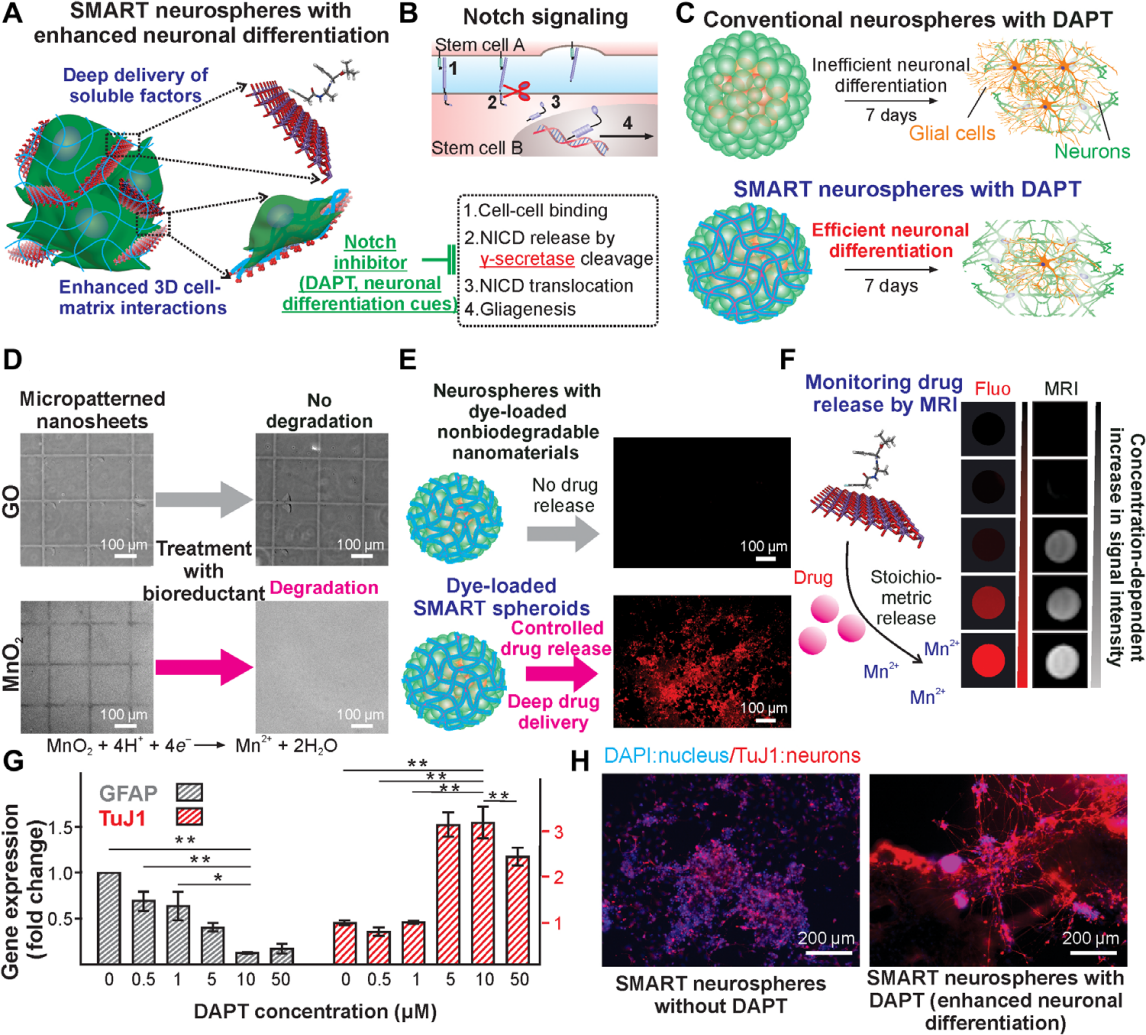
Drug loading and biodegradation-mediated release for enhanced cell fate control. (**A** and **B**) Schematic depicting the deep drug delivery of a Notch inhibitor (Notch-i, DAPT) and its effect on the Notch pathway by release from the embedded nanosheets. (**C**) This leads to an enhanced and efficient neuronal differentiation compared to conventional spheroids, which are hindered by high Notch signaling and lateral inhibition of neurogenesis. (**D** and **E**) Comparison of degradable (MnO_2_) and nondegradable (GO) nanomaterials and their effect on drug delivery. Micropatterns of the nanomaterials were made and allowed to degrade via the addition of ascorbic acid resulting in the degradation of the MnO_2_, whereas the GO did not degrade as analyzed by optical microscopy. This also correlated to the formation of spheroids with the nanomaterials loaded with rhodamine B (RhB) as a model drug, where the MnO_2_ condition degraded and released the drug, while GO did not. (**F**) The drug release could be further correlated with the MRI signal of the degradation product Mn^2+^. (**G**) Concentration-dependent neurogenesis by loading various concentrations of Notch-i (DAPT) on the surface of the nanosheets from 0 to 50 μM as assayed by qPCR. Data are means ± SEM; *n* = 4; *P < 0.05, **P < 0.01 by one-way ANOVA. (**H**) Enhanced neuronal differentiation of SMART neurospheres with Notch-i (DAPT) compared to conventional spheroids and SMART spheroids as measured by immunostaining of TuJ1 (red), a neurogenic marker.

We then sought to integrate deep drug delivery, or delivery of drugs homogeneously throughout the 3D tissue, with SMART neurospheres to synergistically induce neuronal differentiation of hiPSC-NSCs (*58*). Biomaterial-mediated controlled drug delivery is considered a promising approach to bridge the gap between in vitro and in vivo microenvironments; however, the drug diffusion barrier in 3D spheroid structures limits their therapeutic outcome substantially (*59*). SMART neurospheres can overcome these barriers by incorporating drugs during the SMART assembly process. Specifically, drugs can first be loaded onto the MnO_2_ nanosheets to form the SMART neurospheres, thereby facilitating their homogeneous distribution throughout the 3D cell assemblies (Fig. 3A). Despite many methods reported for inducing in vitro differentiation of neurospheres into neurons, their translation into in vivo systems is often unsatisfactory. The difficulty of suppressing the inhibitory soluble microenvironment factors in vivo is recognized as one of the major hurdles.

To this end, the delivery of Notch inhibitors N-[N-(3,5-Difluorophenacetyl)-L-alanyl]-S-phenylglycine t-butyl ester (DAPT), is of our topmost interest, as the Notch signaling pathway has been widely associated with the transcriptional induction of gliogenesis, a major neuroinhibitory factor at CNS disease/injury sites (*60*). Other cell-cell interactions such as cadherin signaling can also be explored but are outside the scope of this current work. Hence, DAPT, a small-molecule γ-secretase inhibitor, is loaded onto MnO_2_ nanosheets under physiological conditions [phosphate-buffered saline (PBS), 37°C] due to its high binding energy calculated by our previously established simulation (Fig. 3B) (*46*). DAPT acts by preventing the cleavage of the Notch intracellular domain, which, in turn, represses Notch signaling, leading to a decrease in gliogenesis and an increase in neuronal differentiation. Afterward, SMART neurospheres with homogeneously distributed Notch-i were formed by mixing a solution of hiPSC-NSCs (1 million/ml, in neurobasal medium) and the second solution of DAPT-loaded, laminin-conjugated MnO_2_ nanosheets following the identical protocol as described above. In parallel, SMART neurospheres assembled from MnO_2_ nanosheets conjugated with a fluorescent aromatic molecule, rhodamine B (RhB), were used as a model to investigate the outcome from deep drug delivery due to its molecular similarities (with aromatic rings and residual nitrogen atoms) with DAPT and considering the difficulties of monitoring DAPT in live cells. We hypothesized that an effective neuronal differentiation could be achieved by a controlled release of DAPT inside the core of SMART neurospheres (Fig. 3C).

To verify our hypothesis, we first established the deep drug delivery in drug-loaded SMART neurospheres by directly monitoring the fluorescence from the controlled release of RhB. Because of its strong binding affinity, the release of RhB bound to MnO_2_ nanosheets is mainly based on the biodegradation of MnO_2_ nanosheets in SMART neurospheres. We confirmed this by performing an in-solution biodegradation assay. Specifically, MnO_2_ nanosheets were patterned as grid shapes by soft lithography, and then a solution of a natural bioreductant, ascorbic acid (vitamin C), was applied to the substrate (Fig. 3D). As a control experiment, graphene oxide (GO) nanosheets were patterned and used, because they have a similar 2D nanosheet structure and are nondegradable. While no biodegradation occurred in a control substrate [(GO) grid pattern nonbiodegradable 2D nanomaterial], the substrate composed of MnO_2_ nanosheets disappeared gradually after 1 hour. This correlated with our drug-releasing experiments, where dye-loaded MnO_2_ or GO nanosheets were used to form spheroids, and after 3 days, only the MnO_2_ condition degraded and released the drug, as indicated by the fluorescence from the model drug RhB (Fig. 3, D and E). Furthermore, we found a relatively homogeneous drug diffusion throughout the spheroid body, with a gradual increase of drug concentrations over 3 days, thereby directly supporting our hypothesis that we can achieve a degradation-mediated deep drug delivery by loading drugs on the nanosheets before assembly (fig. S6A). Notably, this is different from a typical drug gradient pattern when incubating conventional spheroids with drugs or an RhB-GO encapsulated spheroid that did not show apparent drug diffusion (fig. S6B). Moreover, due to this unique drug-releasing mechanism, a stoichiometric release of manganese ions and drugs is expected. Differential drug release is triggered by adding different concentrations of bioreductants that trigger the stoichiometric release of T1 MRI active Mn^2+^ and loaded model drug RhB. We could observe an MRI-monitorable drug release desired for in vivo tracking of therapeutic dosages (Fig. 3F). To further characterize the degradation timeline of our SMART spheroid, inductively coupled plasma mass spectrometry (ICP-MS) analysis on the supernatant of the SMART spheroid cultures was performed to measure the concentration of Mn^2+^ ions. We demonstrated that 75% of the nanosheets in our SMART neurospheres were degraded by 6 days (fig. S6C). Collectively, by using a model drug RhB, we showed the biodegradation-mediated and MRI-monitorable deep drug delivery for effectively controlling the soluble microenvironment in SMART neurospheres.

Furthermore, we investigated whether neuronal differentiation could be enhanced using SMART neurospheres with DAPT. We characterized the formation of SMART neurospheres with DAPT by matrix-assisted laser desorption/ionization–time-of-flight mass spectroscopy (MALDI-TOF) with a characteristic peak at 455 (molecular weight to charge ratio) from the DAPT fragment (fig. S7). As a result of the DAPT delivered into the SMART neurospheres, we observed a DAPT concentration–dependent upregulation of neuronal genes (*TuJ1* mRNA) in the SMART neurospheres with an optimal loading concentration range at 5 to 10 μg/ml (Fig. 3G). In addition, down-regulation of astrocyte genes [glial fibrillary acidic protein (*GFAP*) mRNA] was observed (Fig. 3G). Moreover, there is a strong synergy between the deep delivery of DAPT and incorporation of nanomaterial-mediated 3D cell-matrix interactions, as shown by our immunostaining results on cells differentiated from conventional spheroids, SMART neurospheres, and SMART neurospheres with DAPT, with a 2.15- and 3.95-fold enhancement of neuronal protein marker (TuJ1) expression, respectively (Fig. 3H and figs. S4 and S7). In parallel, the axonal growth in the differentiated neurons was also promoted, with an 8.80- and 1.27-fold increase in the SMART neurosphere with the DAPT group compared to conventional spheroids and SMART neurospheres, respectively (Fig. 3H and figs. S4 and S7). In addition to small-molecule delivery, protein delivery was also tested with success by loading the common growth factor, basic fibroblast growth factor (bFGF), on the surface of nanosheets. When spheroids were formed with bFGF-loaded nanosheets and challenged with medium from activated macrophages (inflammatory signals), much greater survival of cells was seen (fig. S8). Therefore, SMART neurospheres showed a promising combination of biodegradation-mediated deep drug delivery of various cargos with 3D cell-matrix interfaces to regulate the neural cell behaviors of hiPSC-NSCs in vitro.

### Applying SMART neurosphere for enhanced stem cell implantation at CNS injury sites

**Fig. 4.**
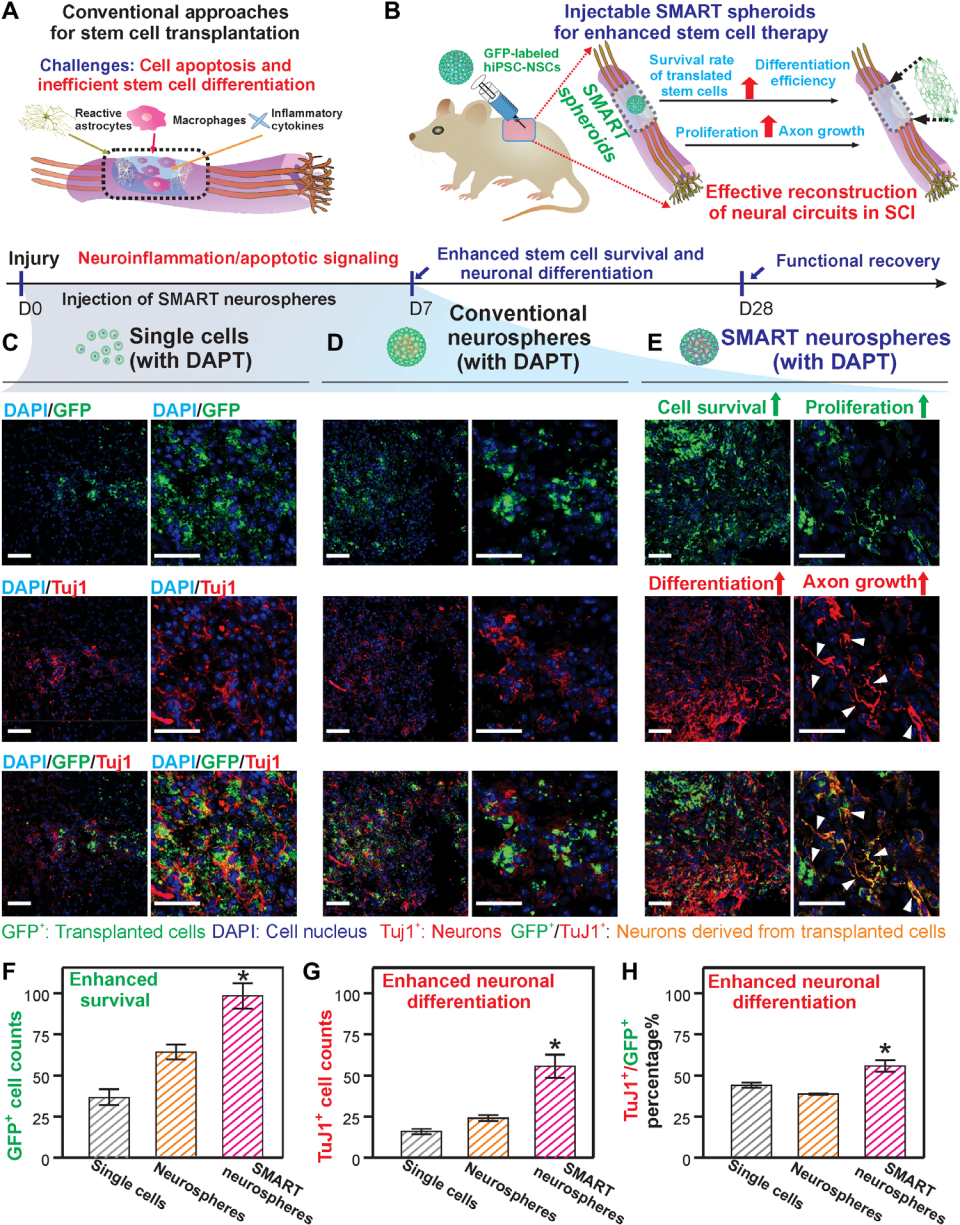
Delivery of SMART neurospheres with DAPT into a murine SCI model. (**A** and **B**) Depiction of the injection of SMART neurospheres with DAPT into a T-10 murine SCI model showing the enhancement in cell survival and differentiation of implanted cells in the SMART neurosphere with DAPT condition compared to single-cell injections with DAPT or conventional neurospheres with DAPT. (**C** to **E**) Immunostaining images of the spinal cord sections (12 μm) showing the GFP (green) signal of implanted cells, DAPI (blue) signal of the cell nuclei, and TuJ1 (red) signal of neuronal cells at 1 week. The number of GFP^+^ cells shows the survival of implanted cells in the injury site, while the GFP^+^/TuJ1^+^ cells show the number of implanted cells that have differentiated into neurons. Scale bars, 50 μm. White arrowheads indicate the formation of processes in the SMART neurosphere group. (**F** to **H**) Quantification of cell survival (GFP^+^ cell counts), the overall number of implanted neuronal cells (GFP^+^/TuJ1^+^ cell counts), and percentage differentiation of implanted cells (GFP^+^/TuJ1^+^ divided by total GFP^+^) showing the clear enhancement of cell survival and differentiation using our SMART neurospheres with DAPT. Images analyzed using ImageJ. Data are means ± SEM; *n* = 3; *P < 0.05 by one-way ANOVA.

With the encouraging results from in vitro stem cell assays, we further evaluated our SMART neurospheres’ potential for improving the survival and neuronal differentiation of hiPSC-NSCs in vivo using a murine SCI model. For cell implantation into the CNS, single-cell injection is currently the clinical standard, despite its extremely low therapeutic efficacy due to poor control over cellular fate in vivo (*5*, *11*). Scaffold-based cell implantation has shown promise for creating favorable microenvironments but often involves invasive surgical procedures and causes long-term immune responses if the scaffolding materials do not degrade in a timely manner (*25*). More recently, spheroids have been considered a unique solution for implanting stem cells; however, an effective neuronal differentiation, which supposedly restores neural circuitries after CNS diseases/injuries, remains a critical hurdle (Fig. 4A).

**Fig. 5.**
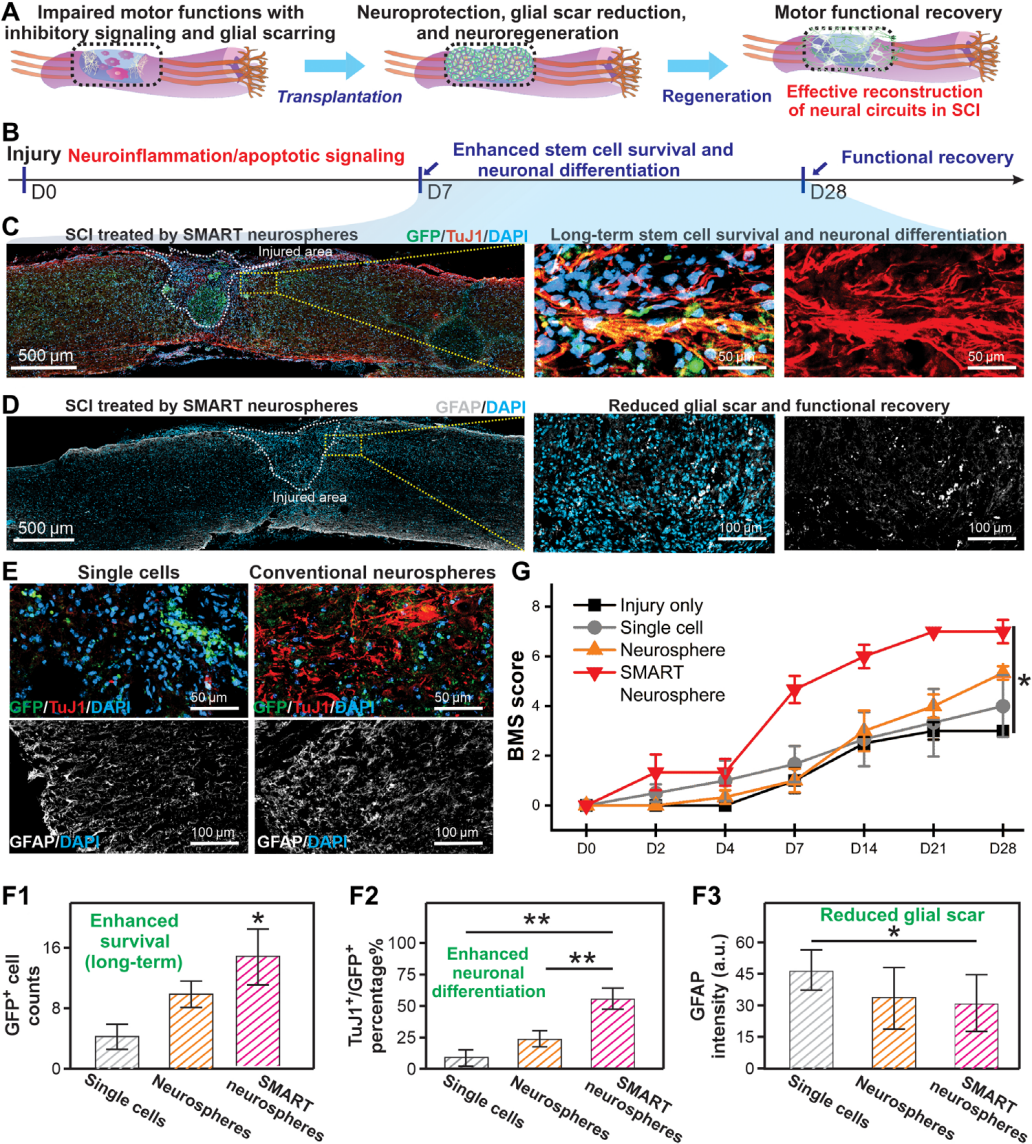
Long-term therapeutic effects of SMART neurosphere treatment. (**A** and **B**) Depiction of long-term survival, differentiation, neuroprotection, and functional recovery after SMART neurosphere injection at a 1-month time point. (**C**) Immunostaining images showing the GFP (green) signal of implanted cells, DAPI (blue) signal of the cell nuclei, and TuJ1 (red) signal of neuronal cells at 1 month after injection of SMART neurospheres. (**D**) Immunostaining images showing the GFAP (white) signal of the glial scar and DAPI (blue) signal of cell nuclei highlighting the injury site and glial scar formation at 1 month after injection of SMART neurospheres. (**E**) Immunostaining images showing the GFP (green) signal of implanted cells, DAPI (blue) signal of the cell nuclei, TuJ1 (red) signal of neuronal cells, and GFAP (white) signal of the glial scar at 1 month after injection of single cells or neurospheres, respectively. (**G**) BMS scoring data showing the functional recovery of animal walking over 4 weeks. Data are means ± SEM; *n* = 3; *P < 0.05 by one-way ANOVA. (**F1** to **F3**) Quantification of cell survival (GFP^+^ cell counts), percentage differentiation of trasplanted cells (GFP^+^/TuJ1^+^ divided by total GFP^+^), and reduction in glial scar intensity showing the clear enhancement of cell survival, differentiation, and reduction in glial scar formation using our SMART neurospheres with DAPT at 1 month. Images analyzed using ImageJ for (F1) and (F2). Images analyzed with Nikon Elements automatic fluorescence detection module. Data are means ± SEM; *n* = 3; *P < 0.05, **P < 0.01 by one-way ANOVA. a.u., arbitrary units.

Addressing the aforementioned challenges, SMART neurospheres, encompassing the advantages from both scaffold-based and scaffold-free methods, may provide a means for injectable stem cell implantation by effectively controlling the soluble/insoluble microenvironment in vivo. To prove this, we created a murine hemisection SCI model at thoracic levels T8 to T10 and then implanted NSCs at the injury sites using SMART neurospheres with DAPT. As controls, single-cell suspensions and conventional neurospheres without any nanomaterials were also injected into the SCI sites with the same total cell numbers (1 million cells per animal) and the same DAPT concentration (10 μM) (Fig. 4B). We used hiPSC-NSCs to be consistent with our in vitro stem cell assay. Furthermore, as SCI sites typically contain highly heterogeneous cell types, hiPSC-NSCs were genetically fluorescent labeled with a green fluorescent protein (GFP) before injection through plasmid transfection using Lipofectamine and then selected for using puromycin to reliably track their fates after implantation. Seven days post-injection (DPI), we found a significantly higher cell survival in the experimental condition (SMART neurospheres with DAPT), with a nearly 2.69- and 1.55-fold enhancement compared to single-cell injection and control neurosphere groups, respectively (quantified by counting the number of GFP-labeled hiPSC-NSCs) (Fig. 4, C to F). This result is consistent with our conclusions in the in vitro stem cell survival assay (fig. S8) and could be supported by the 3D cell-matrix (compared to control neurospheres) and cell-cell (compared to the single-cell injection) interactions. Furthermore, an improved neuronal differentiation efficiency is also found in the SMART neurosphere group (with DAPT), with an 11.6 and 17.2% increase compared to single-cell injection and control neurosphere groups, respectively, which is quantified by the percentage of dual-positive cells (GFP^+^/TuJ1^+^, an early neuronal marker) to implanted cells (GFP^+^) (Fig. 4, G and H). Many hiPSC-NSC–derived neurons show apparent axonal growth with cellular processes that are only observed in the SMART neurosphere group (with DAPT). The formation of processes from neurons is a crucial sign of integration into host neural circuitries (*61*). The extent of axon sprouting was quantified and demonstrated that the SMART neurosphere group achieved four times longer axon outgrowth compared to control conditions (fig. S9). Although several stem cell implantation studies in SCI models have shown similarly high neuronal differentiation rates and the formation of axonal growth from the differentiated neurons, an immunosuppressant has typically been administered to ensure a relatively friendly microenvironment at SCI sites. To confirm our results at a longer-term time point, we also assayed the survival and differentiation of our implanted cells 1 month after injury (Fig. 5, A and B). We demonstrated an enhanced cell survival with an approximately 1.5- and 3.3-fold increase in cell counts in the SMART neurosphere group (with DAPT) compared to the neurosphere group and single-cell injection groups, respectively. In addition, this correlated with an enhanced differentiation of the implanted cells, with the experimental group having 55% differentiation into neurons compared to 23% in the neurosphere group and 9% in the single-cell injection group (Fig. 5, C, E, and F1 and F2). This interesting result shows that, even at 1 month, both enhanced survival and differentiation of stem cells could be observed in our SMART neurosphere as compared to control conditions. To understand the effect of this enhanced survival and differentiation of our SMART neurospheres on SCI, we looked at two markers that correlate with the severity of injuries. We first measured the glial scar formation after injury by staining an astroglial protein marker GFAP. We observed a reduced glial scar intensity in our experimental condition by about 26% compared to the single-cell condition bordering the injury site (Fig. 5, D, E, and F3). Next, we looked at the marker NeuN, which labels the endogenous neurons in the spinal cord’s ventral horn. We studied the survival of these neurons after injury by counting NeuN^+^ cells around the injury site. We observed a 1.7- and 3.6-fold increase in NeuN^+^ cells in the experimental condition compared to neurosphere injections and single-cell injections, respectively (fig. S10). These results demonstrate the reduction in scar formation and neuroprotective effect of our SMART neurospheres compared to traditional neurospheres and single-cell injections, which may be attributed to the secretion of neurotrophic factors from the hiPSC-NSCs and the release of DAPT from the spheroids. Last, these positive results strongly correlated with an increase in Basso Mouse Scale (BMS) scoring, in which the SMART neurosphere conditions showed an average BMS score of 6.5 at 1 month, while the neurosphere group, single-cell group, and an injury only group showed average scores of 4.25, 4, and 3, respectively (Fig. 5G and movie S1). This demonstrates a faster rate of recovery and improved functional recovery in the experimental group compared to control groups at a 1-month time point. Here, we provide definitive proof that the robustly enhanced cell survival and neuronal differentiation of NSCs could be effectively realized even without any immunosuppressants by using our SMART neurosphere technology, and this enhancement correlated with a reduction in glial scar formation, increase in endogenous neuron survival, and functional recovery of animal walking at a 1-month time point.

Moreover, as the nanomaterials (MnO_2_ nanosheets) incorporated in the SMART neurospheres effectively biodegrade by cell-secreted bioreductants, we hypothesized that there would be minimal systemic cytotoxicity from the implantation of SMART neurospheres. Hence, we first confirmed the timely in vivo biodegradation of MnO_2_ nanosheets in the SMART neurospheres, as supported by the apparent disappearance of dark-colored materials 7 DPI. As the biodegradation of MnO_2_ nanosheets is associated with the production of Mn^2+^ ions, we further performed urine and blood analysis over a 4-week period and verified initial biodegradation at 7 DPI (supported by the 2.1-fold increase of manganese levels in urine samples) and complete degradation at 28 DPI (manganese levels returned to normal) (fig. S11). This was also verified by the visual disappearance of the dark MnO_2_ scaffold in the spinal cord after 1 week (fig. S11C). Rapid biodegradation is desired because the initial 1 to 2 weeks after SCI often contains the most hostile microenvironments that require the most notable support from our nanomaterials. Meanwhile, long-term immune reactions to exogenous materials typically occur at 1 month after implantation, according to the literature (*59*). Next, we also studied whether there is any systemic cytotoxicity from the implantation of our SMART neurospheres. Although manganese exists in many enzymes (e.g., MnSOD) and is an essential element for human metabolism, a burst administration of manganese at high dosages has been shown to induce toxicities. Therefore, we analyzed the effects of SMART neurosphere implantation on major organs responsible for metabolism and detoxification, including the kidneys, liver, heart, lungs, and spleen. As a control, mice implanted with single-cell injections under identical conditions were used. There was no long-term accumulation of manganese ions in any of the organs. In addition, histology was performed on the liver and kidneys, which are the two major organs involved in metabolism and detoxification in the body. It was shown that none of the slices from these major organs showed any clear signs of tissue damage in the SMART neurosphere group compared to the control group (fig. S11 and table S2). Together, our in vivo stem cell implantation assay and toxicity assay indicate the enormous potential of our SMART neurosphere technology for improving stem cell therapy with minimal systemic toxicities.

The effective treatment of devastating diseases and disorders of the CNS is of the utmost importance, but currently, there is a lack of viable treatment strategies for disorders such as SCI. While stem cell–based cell replacement therapies are very promising, they are hindered by the survival and control of cell fate after implantation (*5*, *11*). Here, we developed a novel approach that can control cell fate on two levels—both through insoluble physical interactions and soluble drug delivery. By using our hybrid cell spheroids, we can achieve effective control of cell-cell and cell-matrix interactions as well as deep delivery of small molecules and growth factors to aid in the survival and differentiation of stem cells both in vitro and in vivo. We applied this developed technology to the treatment of an SCI model and showed an increase in cell survival, differentiation, and enhanced neuronal behaviors, such as axon growth, compared to control conditions, showing the therapeutic potential of our SMART spheroids. For this project, we used NSCs and neuronal differentiation as proof-of-concept demonstrations. Our system is very adaptable for any cell and differentiation type and can be applied to treat a variety of diseases and disorders. In addition, this opens the potential for creating spheroids encompassing a wide array of cell types simultaneously, which can have broad implications in organoid development and disease modeling by creating more reproducible and biomimetic 3D cell architectures. Because of this, we believe that our technology platform is an ideal candidate for improving many other types of cell therapies that require high cell survival and effective control of cell fate, making it useful not only for treating SCI but also for various other diseases and disorders.

## MATERIALS AND METHODS

### Synthesis and characterization of MnO_2_ and GO nanosheets

Synthesis of the MnO_2_ nanosheets was based on a previously reported protocol with minor modifications (*20*, *62*). Briefly, 2.2 g of tetramethylammonium pentahydrate (TMAOH·H_2_O; Alfa Aesar) was dissolved in 20 ml of 3 weight % (wt %) H_2_O_2_ (Sigma-Aldrich) and vortexed. In addition, 0.594 g of MNCl_2_·H_2_O (Sigma-Aldrich) was dissolved in 10 ml of deionized water by sonication. The TMAOH solution was then dissolved in the MnCl_2_ solution rapidly under stirring at 1200 rpm. The solution was stirred overnight at 600 rpm overnight and then centrifuged at 2000*g* for 5 min to obtain the bulk δ-MnO_2_. The product was then washed with water and ethanol four times by mixing and centrifuging and dried in an oven under ambient conditions. The product was then added to deionized water to a concentration of 10 mg/ml and sonicated for 10 hours. Last, the solution was centrifuged at low speeds (8801*g*) for 10 min to remove aggregates. The nanosheets were diluted to 10 μg/ml for transmission electron microscopy (TEM) (80 kV on a Philips CM12 with an Advanced Microscopy Techniques (AMT) digital camera model XR111). The hydrodynamic size and zeta potential of the nanosheets were measured using a Nano Zetasizer dynamic light scattering system at a detection angle of 90°. GO was also synthesized on the basis of a previous report (*19*), where 1 g of graphite (Bay Carbon) was preoxidized in the mixture of sulfuric acid (98%; Sigma-Aldrich), phosphorus pentoxide (Sigma-Aldrich), and potassium persulfate (Sigma-Aldrich) at 80°C overnight. It was then washed with water, dried, and reacted with sulfuric acid and potassium permanganate. The solution was then quenched with hydrogen peroxide and then purified with hydrochloric acid (Sigma-Aldrich) and washed extensively with water. The GO was then exfoliated by tip sonication (Branson) and purified by centrifugation at 17,000*g* for 45 min.

### Protein and drug loading on MnO_2_ nanosheets

For the loading of proteins on the surface of nanosheets, we used the common ECM protein natural mouse laminin (Sigma-Aldrich). We made a solution of laminin in PBS (Thermo Fisher Scientific) at a concentration of 20 μg/ml and mixed it with the MnO_2_ nanosheet solution at a concentration of 3 mg/ml at a final ratio of 5 μg of laminin for every 100 μg of MnO_2_. This was further diluted in Dulbecco’s modified Eagle’s medium (DMEM)/F12 to reach a final MnO_2_ concentration of 1 mg/ml. The final solution was allowed to incubate overnight at 37°C and then centrifuged to remove excess laminin protein and redispersed in DMEM/F12 and sonicated before use to form spheroids. We adopted a similar strategy for loading drugs onto the surface of nanosheets for degradation and characterization studies. First solutions of DAPT (Tocris, catalog number 2634) and RhB (Alfa Aesar, catalog number A13572) were diluted to solutions of 20 mM and mixed with our MnO_2_ solution to a final concentration of 50 μM. This solution was allowed to react overnight at 37°C before washing thoroughly, at least six times with PBS by centrifugation to remove the excess drug. To confirm the binding of DAPT onto the surface of MnO_2_, MALDI-TOF (Bruker, Ultraflex) was used on the basis of the Na^+^-DAPT peak at 455 (molecular weight–to–charge ratio).

### NSC culture and spheroid formation

hiPSC-derived NSCs were derived from hiPSCs (WT126 clone 8 and WT33 clone 1) (*63*) and grown in proliferation medium containing DMEM/F12 (Invitrogen) supplemented with B27 (Invitrogen), N2 (STEMCELL Technologies), and bFGF (20 ng/ml) (Invitrogen). Cells between passages 5 and 10 were used for all experiments. Total viable cell numbers were assessed by counting the single-cell suspensions of hiPSC-NSCs with an ADAM-MC2 cell counter (NanoEnTek, Seoul, Korea) according to the manufacturer’s instructions. Briefly, to form spheroids, a solution of NSCs (hiPSC-NSCs, 1 million cells/ml, in neural growth medium) was mixed with our previously formed solution of laminin-loaded MnO_2_ in a 1.7-ml Eppendorf tube and pipetted vigorously. The formation of aggregates was visible within 15 min; however, the solution was allowed to incubate at 37°C for a minimum of 4 hours before being transferred to tissue culture plates (Corning) for future studies. For light microscopy studies, cells were mixed with varying concentrations of laminin-loaded MnO_2_ and formed as previously described and then imaged using the phase contrast mode using a Nikon Eclipse Ti-E microscope. Besides, after 48 hours of culture, cell viability was assayed using a standard PrestoBlue assay (10% volume ratio as compared to cell medium; Thermo Fisher Scientific, catalog no. A13261) following the manufacturer’s instructions. Furthermore, for SEM studies, cells were plated on glass slides and cultured as previously described and then chemically dried using serial increments of ethanol and hexamethyldisilazane (Thermo Fisher Scientific). Last, cells were removed from all solutions and air-dried overnight before sputter coating with a 20-nm-thick gold film. Cells were then visualized using the FE-SEM (Zeiss with Oxford EDS). For FAK inhibitor studies, FAK inhibitor 14 (Sigma-Aldrich) was treated to cells 1 hour before spheroid formation. After treatment, spheroids were made following the previously mentioned protocol.

### In vitro differentiation of SMART spheroids

For in vitro differentiation studies, SMART spheroids were generated as previously described. Briefly, the mixtures of hiPSC-NSCs and laminin-coated nanosheets were mixed with 1 million cells/ml and varying concentrations of nanosheets from 0 to 100 μg/ml and allowed to incubate to form the various spheroids. Cells were then transferred to 24-well plates and allowed to differentiate by removing the growth factor bFGF from the neural proliferation medium. A half-medium change was performed every day for 7 days, at which point the cells were either fixed using 10% formalin (Sigma-Aldrich) for immunocytochemistry or lysed for PCR experiments using TRIzol (Thermo Fisher Scientific). For immunocytochemistry, cells were fixed and stained using Hoechst (1:100 dilution, 0.2 mM; Thermo Fisher Scientific, catalog number 33346), a neuronal marker (TuJ1; 1:500 dilution; Cell Signaling Technology, catalog number 4466), and a mature neuronal marker (MAP2; 1:200 dilution; Cell Signaling Technology, catalog number 4542S). The cells were then imaged using a Nikon Eclipse Ti-E microscope, and all images were analyzed for percent differentiation using the ImageJ software, where nuclei were counted and cells were studied for whether they were TuJ1 positive. The axon length was studied using the NeuronJ software in ImageJ for axon tracing using the TuJ1 signal. For qRT-PCR, the TRIzol samples were collected and reverse-transcribed using the SuperScript III First-Strand Synthesis System (Life Technologies). The complementary DNA (cDNA) was then used for qPCR using a StepOnePlus RT-PCR system (Applied Biosystems), a Power SYBR Green PCR master mix (Applied Biosystems), and primers specific to each target gene (*TuJ1*, *FAK*, and *Notch2*). All samples were normalized to glyceraldehyde-3-phosphate dehydrogenase (*GAPDH*) gene expression.

### Size control of SMART spheroids

Hydrogel microwell arrays were fabricated using a previously described protocol using a polydimethylsiloxane (PDMS) stamp (*64*). Briefly, the photomasks were designed in AutoCAD (Autodesk, USA), then printed on a photomask and transferred onto silicon wafers (Wanxiang Silicon-Peak Electronics Co., China) using SU-8 negative photoresist (MicroChem Corp., USA), and developed according to the manufacturer’s instructions. PDMS prepolymer solution (10:1, monomer:curing agent; Sylgard 184, Dow Corning Corp., USA) was poured onto the silicon molds, and air bubbles were removed in a vacuum chamber for 30 min followed by curing in an oven at 85°C for 2 hours. Afterward, we generated the hydrogel microwell arrays by pouring a solution of 10% (w/v) polyethylene glycol diacrylate with a molecular weight of 700 and 1% photoinitiator onto a 3-(trimethoxysilyl) propyl methacrylate–treated glass followed by pressing under the PDMS stamp and ultraviolet light exposure. Spheroids were then formed using the aforementioned method and seeded into the microwells in 24-well plates. After seeding, cells were allowed to incubate for 2 hours at 37°C before wiping away cells that were not located inside the microwells. Cells were then allowed to incubate at 37°C overnight before being transferred by agitation and pipetting into fresh 24-well plates. These cells were then allowed to differentiate for 7 days by removing bFGF and were fixed and imaged as previously described for Hoechst and Tuj1.

### MnO_2_ degradation studies

To study the degradation of the MnO_2_ nanosheets, MnO_2_ and GO nanosheets were micropatterned on indium tin oxide (ITO) glass using soft lithography. Briefly, PDMS stamps were created by pouring PDMS prepolymer solution (10:1, monomer:curing agent; Sylgard 184, Dow Corning Corp., USA) onto the silicon molds of a grid pattern, and air bubbles were removed in a vacuum chamber for 30 min followed by curing in an oven at 85°C for 2 hours. The PDMS stamps were then dipped in a solution of dye-loaded MnO_2_ or GO and pressed on the surface of ITO glass for 10 min under pressure. The ITO substrates were then imaged using the optical microscope to visualize the micropattern. Afterward, substrates were treated with ascorbic acid (25 g/ml) and visualized again using an optical microscope to see the degradation of the MnO_2_ pattern, but the GO patterned did not.

### Degradation-mediated drug release studies

Concurrently, spheroids were formed as previously described using both dye-loaded MnO_2_ and GO. Cells were then transferred to a 24-well plate and imaged daily for the fluorescent signal. The samples that used MnO_2_ nanosheets saw a day-dependent increase in fluorescent signal, whereas the GO samples showed little to no fluorescent signal. To correlate the release of drugs with the MRI signal generated by the degradation product, Mn^2+^ varying concentrations of dye-loaded MnO_2_ nanosheets were fully degraded using ascorbic acid. The samples were then measured using both fluorescent microscopies to quantify drug release and MRI imaging (Aspect’s M2TM Compact High-Performance MRI, 1T) to quantify manganese ion content and MRI signal. As expected, the correlation could be seen between the fluorescent drug release and the MRI signal.

### DAPT-mediated neurogenesis

To study the effect of DAPT-loaded MnO_2_ nanosheets on neurogenesis of SMART spheroids, we first loaded varying concentrations of DAPT on the surface of the MnO_2_ nanosheets at concentrations from 0 to 50 μM as previously described. These drug-loaded nanosheets were then used to form spheroids, and then cells were transferred to 24-well plates and allowed to differentiate for 7 days by bFGF withdrawal. Cells were then fixed with formalin or lysed with TRIzol, as previously described. Cells that were fixed were stained and imaged for a nuclear marker (Hoechst) and a neuronal marker (TuJ1). The cells were then imaged using the Nikon Eclipse Ti-E microscope, and all images were analyzed for percent differentiation using the ImageJ software, where nuclei were counted and cells were studied for whether they were TuJ1 positive. Besides, the axon length was studied using the NeuronJ software in ImageJ for axon tracing using the TuJ1 signal. For qRT-PCR experiments, the TRIzol samples were collected and reverse-transcribed using the SuperScript III First-Strand Synthesis System (Life Technologies). The cDNA was then used for qPCR using a StepOnePlus RT-PCR system (Applied Biosystems), a Power SYBR Green PCR master mix (Applied Biosystems), and primers specific to each target gene (*TuJ1* and *GFAP*). All samples were normalized to *GAPDH* gene expression.

### In vitro inflammatory survival assay

To study the survival of our hiPSC-NSCs when challenged with inflammatory molecules, we first cultured THP-1 monocytes and differentiated them into macrophages by stimulating them with lipopolysaccharide. We then harvested the medium from the macrophages and cocultured traditional neurospheres or our SMART neurospheres with conditioned medium at a 10:1 ratio with basal medium. We allowed the cells to incubate for 24 hours and then assayed them using LIVE/DEAD staining (Thermo Fisher Scientific, catalog no. L7010) to study the cell survival under inflammatory conditions.

### In vivo delivery of SMART spheroids assay

All experiments were approved by the Institutional Animal Care and Use Committee (IACUC) and the Institutional Biosafety Committee at Rutgers University. All animal work was conducted in compliance with the National Institutes of Health *Guide for the Care and Use of Laboratory Animals*. All experiments were done in accordance with the Rutgers IACUC standards under protocol 999900038.

For the in vivo experiments, a T8-T10 hemisection model was used with 8-week-old C57BL6 mice. Mice were randomly selected for each experimental group with *n* = 3 per group. The dorsal fur was shaved off 1 day before the surgery. Next, an oxygen-enriched 5% isoflurane chamber was used to anesthetize the animals and then maintained at 2% isoflurane for the duration of the surgery. For the hemisection, a laminectomy of the T10 vertebral bone was performed to expose the spinal cord. Next, the dorsal blood vessel was cauterized, and the spinal cord was cut from the midline toward the left using a #10 scalpel.

Next, SMART spheroids or control conditions were injected into the spinal cord using 1 million cells per animal. For in vivo studies, cells were labeled with a GFP plasmid for cell tracking purposes. For each group, cells, DAPT, laminin, and bFGF were injected into the spinal cord. For the single-cell conditions, cells were monodispersed and injected with the three other components in solution. For the neurosphere condition, cells were preaggregated into spheroids by culturing overnight in nonattachment dishes and then injected in solution with the three other components. In the SMART spheroid condition, all three components were loaded onto the surface of the MnO_2_ nanosheets, formed into spheroids with the cells, and then injected. For all conditions, 1 million cells were used with 10 μM DAPT, 5 μg of laminin, and 1 μg of bFGF.

At 1 and 4 weeks after injury, animals were euthanized, and organs, including the spinal cord, were harvested using dissection. They were washed in 1× PBS and fixed with 4% (w/v) paraformaldehyde for 24 hours. Fixed tissues were then rewashed and cryopreserved in 30% (w/v) sucrose for 48 hours. Next, the spinal cord tissue was embedded in cryopreserving medium (Tissue-Tek OCT compound) and kept frozen at −80°C for further processing. Spinal cord tissue was sectioned at 12 μm thickness and then stained using Hoechst, TuJ1 (previously described), GFAP (Invitrogen, catalog number PAS-16291), and NeuN (BioLegend, catalog number 834501) antibodies and imaged on a confocal microscope (Zeiss LSM 800). Cell counting for percentage differentiation analysis was performed on ImageJ. Intensity analysis for glial scar formation was performed using the Nikon Elements software automatic fluorescence detection module. One-way analysis of variance (ANOVA) was used for multigroup analysis. Data are means ± SD; *n* = 3; *P < 0.05, **P < 0.01 with Tukey’s post hoc analysis. In addition, urine and blood for the animals were collected from animals at 1- and 4-week time points by harvesting bedding and soaking it in ultrapure water or extraction immediately after sacrificing, respectively.

### In vivo ICP-MS analysis and tissue histology

For ICP-MS analysis, the blood and urine were diluted in aqua regia and water and analyzed for content of common ions, including Mn^2+^ ions. For ICP-MS of organs, organs were first cut and ground using a scalpel and mortar and pestle. Ground-up samples were then dissolved in aqua regia and filtered to remove any remaining particulates and then analyzed for common ions, including Mn^2+^. For tissue histology studies, all samples were sent to the Rutgers Molecular Imaging Center for processing and analysis, where a board-certified pathologist analyzed the tissue sections and found no noticeable differences between experimental and control conditions.
