# Supplementary material for: Hybrid SMART spheroids to enhance stem cell therapy for CNS injuries
Source: Sci Adv. 2021 Sep 29;7(40):eabj2281. doi: 10.1126/sciadv.abj2281 (PMC8480929; doi:10.1126/sciadv.abj2281)
Supplement: Supplementary file 1 — Figs. S1 to S11 Tables S1 and S2 Legend for movie S1 [file sciadv.abj2281_sm.pdf]

Science Advances
AAAS

Supplementary Materials for

Hybrid SMART spheroids to enhance stem cell therapy for CNS injuries

Christopher Rathnam, Letao Yang, Sofia Castro-Pedrido, Jeffrey Luo, Li Cai, Ki-Bum Lee*

*Corresponding author. Email: kblee@rutgers.edu

Published 29 September 2021, *Sci. Adv.* **7**, eabj2281 (2021)
DOI: 10.1126/sciadv.abj2281

The PDF file includes:

Figs. S1 to S11
Tables S1 and S2
Legend for movie S1

Other Supplementary Material for this manuscript includes the following:

Movie S1

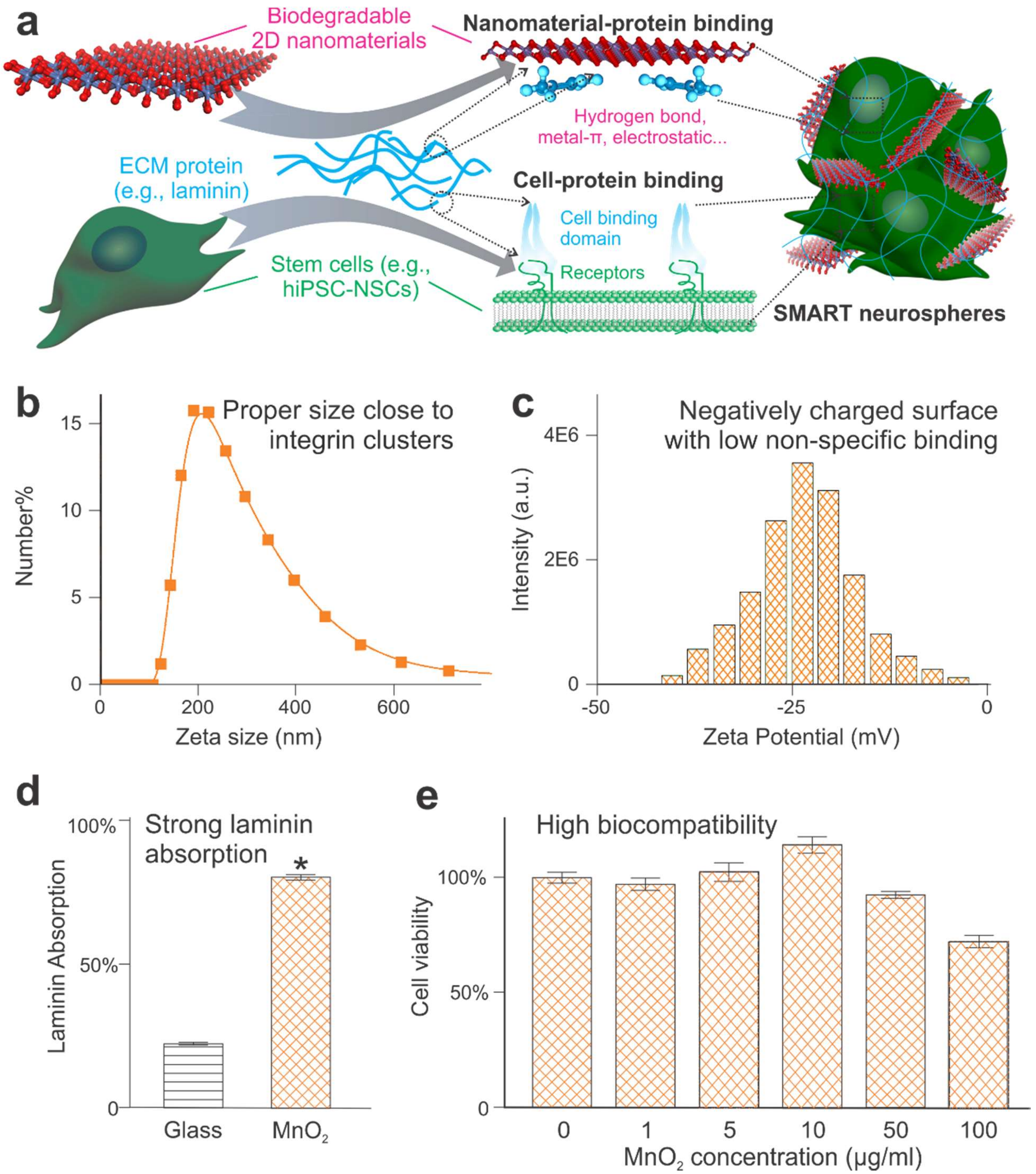

SUPPORTING FIGURE 1: Synthesis and properties of manganese dioxide nanosheets. (a) Overall assembly of the SMART neurospheres requires the proper size, protein binding, and biocompatibility of the nanosheets. **b-e**, Zeta size and Zeta potential of synthesized MnO_2 nanosheets (**b-c**). Protein loading on the surface of MnO_2 nanosheets as measured using a BCA Assay Data are mean ± s.t.d. n = 3. *p <.01 by student's t-test (**d**). Viability of hiPSC-NSCs when formed into neurospheres after 48 hours as measured by a Presto Blue Assay (**e**). Data are mean ± s.t.d. n = 3.

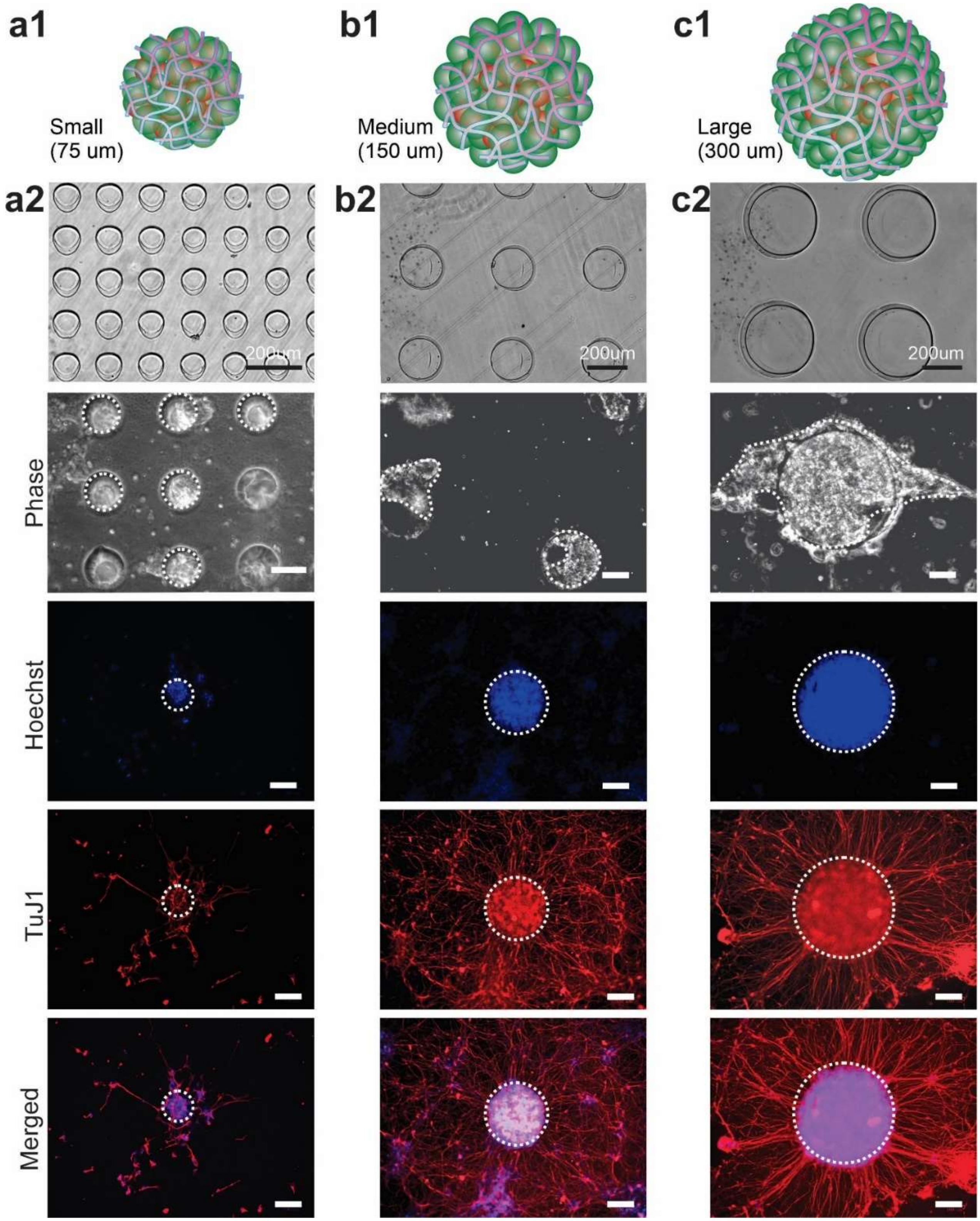

SUPPORTING FIGURE 2: Size control of SMART Spheroids. (a1, b2,c1) Size control of SMART Spheroids using PEG hydrogels. Hydrogels of 3 sizes a2) 75, b2) 150, c2) 300 μm were used to template the growth of the spheroids, which were then transferred into 24 well plates and differentiated for 7 days. Scale bars: 100 μm.

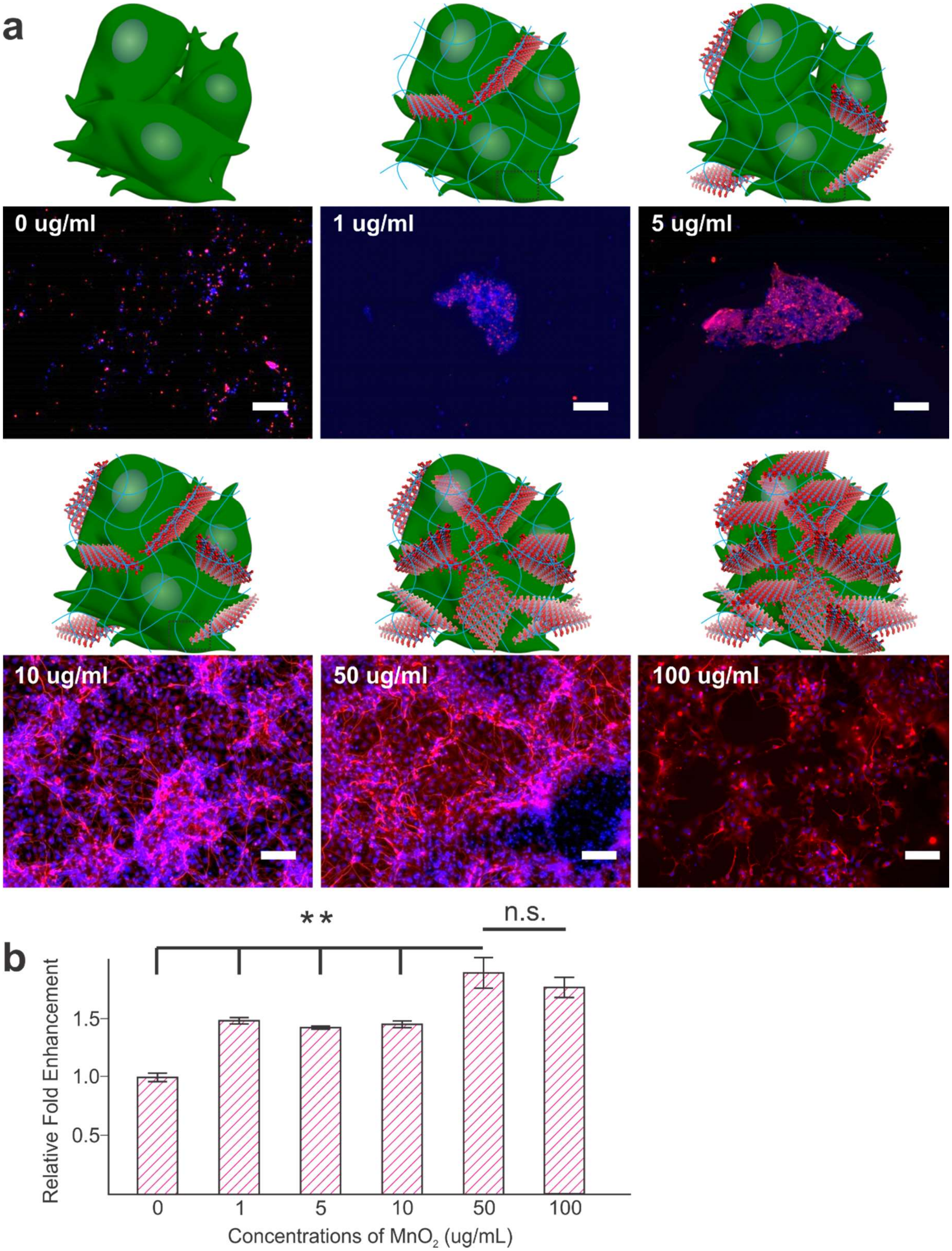

SUPPORTING FIGURE 3: Enhanced neurogenesis via manganese dioxide nanosheets. (a) Nanosheet concentration-dependent enhancement of neurogenesis as characterized by immunostaining of neuronal marker Tuj1(red) and nuclear marker Hoechst (blue). (b) In addition, a qRT-PCR analysis was performed for the neuronal marker TuJ1. Data are mean ± sem n = 3, * $P < 0.05$ ** $P < 0.01$ by one-way ANOVA. Scale bars: 100 μm.

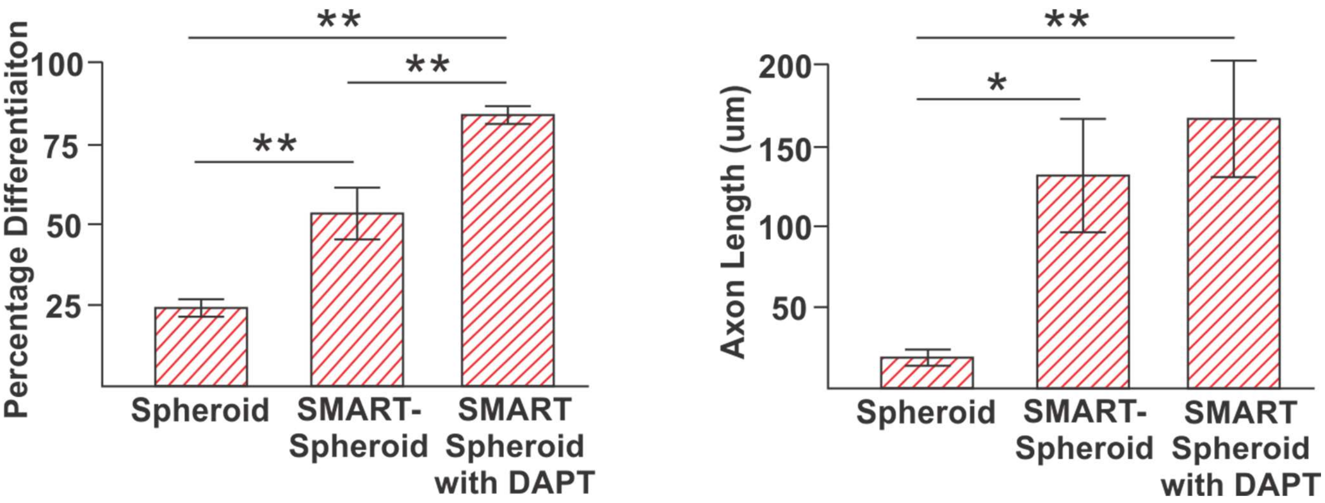

SUPPORTING FIGURE 4: Quantification of axon length and percentage differentiation comparing traditional neurospheres to our SMART neurosphere and SMART DAPT conditions using 10uM DAPT. Data are mean ± sem n = 4, * P < 0.05 ** P < 0.01 by one-way ANOVA

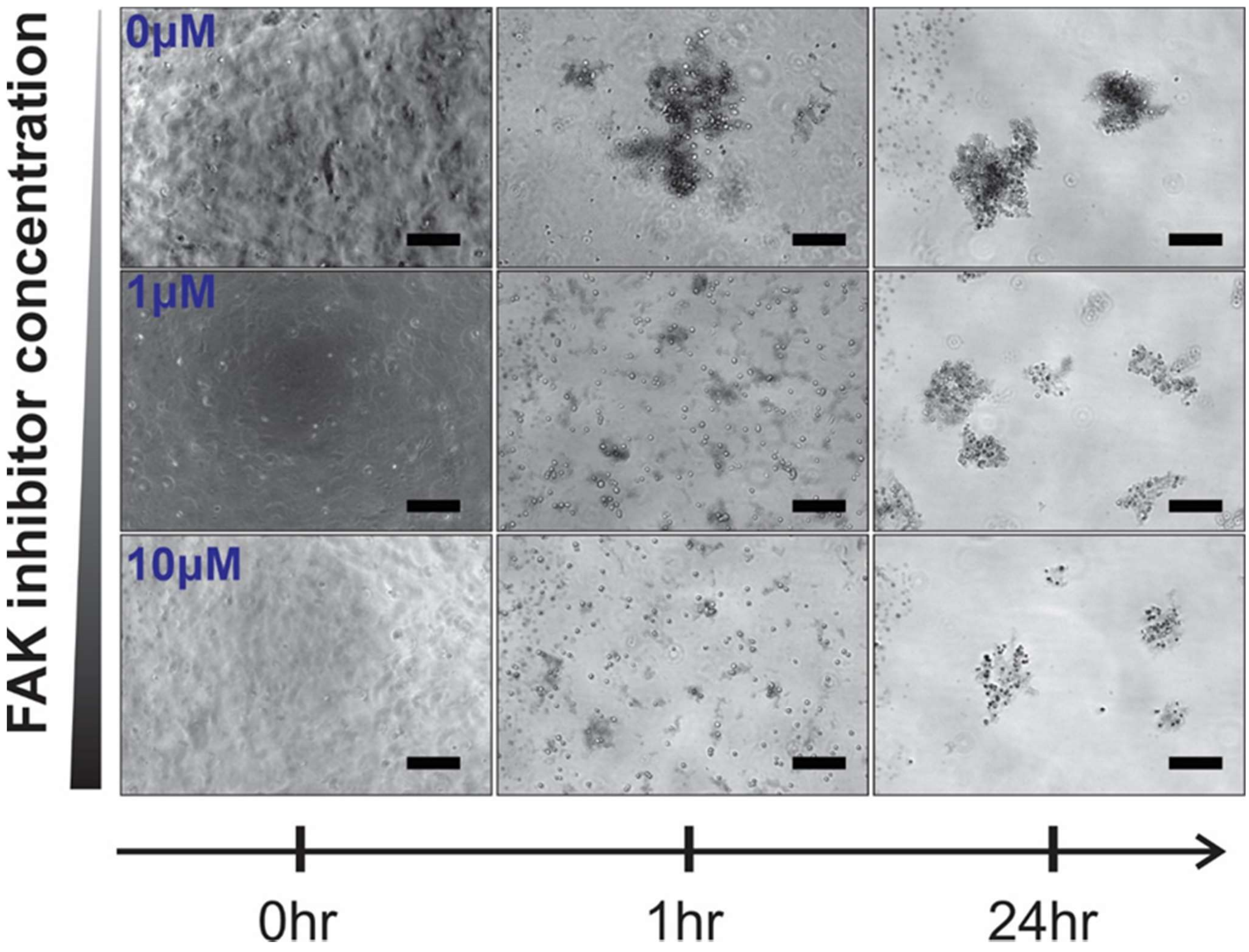

SUPPORTING FIGURE 5: The formation of spheroids with increasing concentration of a focal adhesion kinase inhibitor at 1 and 24 hrs. Spheroids with 1 and 10 μM treatment of the FAK inhibitor shows delayed and impaired spheroid formation resulting in a lack of formation of spheroids or very loosely packed small cell aggregates. Whereas, control spheroid assembled in <1hr and showed more condensed tightly packed spheroids owing to focal adhesion formation and signaling. Scale bars: 100 μm

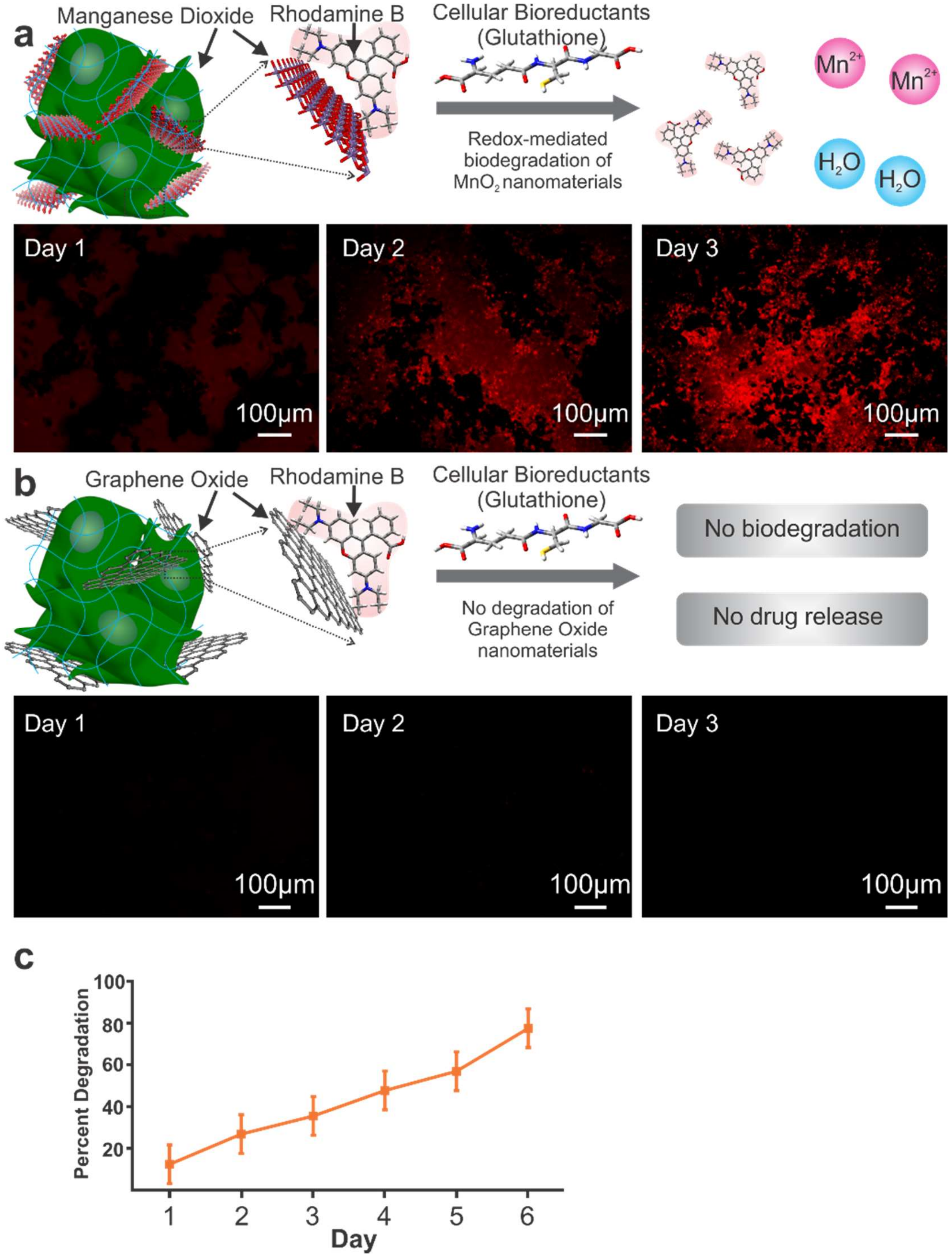

SUPPORTING FIGURE 6: Degradation mediated drug release in SMART Spheroids. Time-dependent increase in drug release from RhB loaded nanosheets in SMART Spheroids (**a**) and graphene oxide-derived spheroids (**b**) assembled from hiPSC-NSCs. Spheroids were cultured in 24 well plates for up to 3 days and the fluorescent signal was measured each day using fluorescent microscopy. Due to the toxicity of the model drug RhB at high concentrations after Day 3, cells become unhealthy. (**c**)ICP-MS was used to quantify percent degradation of the scaffold in SMART Spheroids without dye loading by collecting the supernatant and measuring Mn^{2+} concentration. This was plotted in a day-dependent manner.

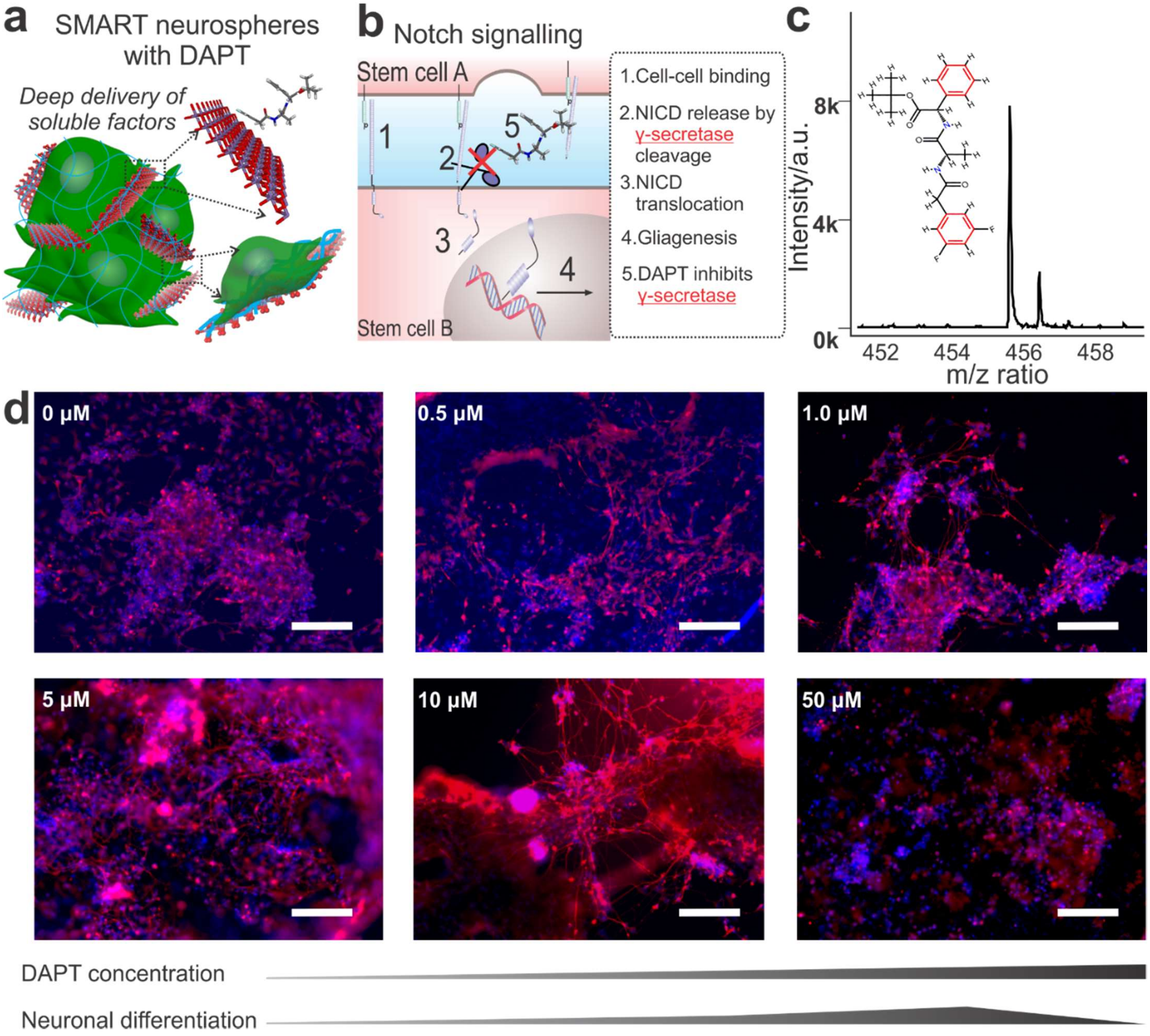

SUPPORTING FIGURE 7: Enhanced neurogenesis via nanomaterial-based drug delivery. (a,b) Notch inhibitor, DAPT, acts as a potent neurogenic drug when delivered deeply to stem cells. (**c**) Maldi-ToF data showing the loading of DAPT on the surface of MnO_2 nanosheets. (**d**) Concentration-dependent enhancement of neurogenesis as characterized by immunostaining of neuronal marker Tuj1(red) and nuclear marker Hoechst (Blue). Optimal enhancement of percent differentiation and axon length was found at 5-10 μM concentration. Scale bars: 200 μm.

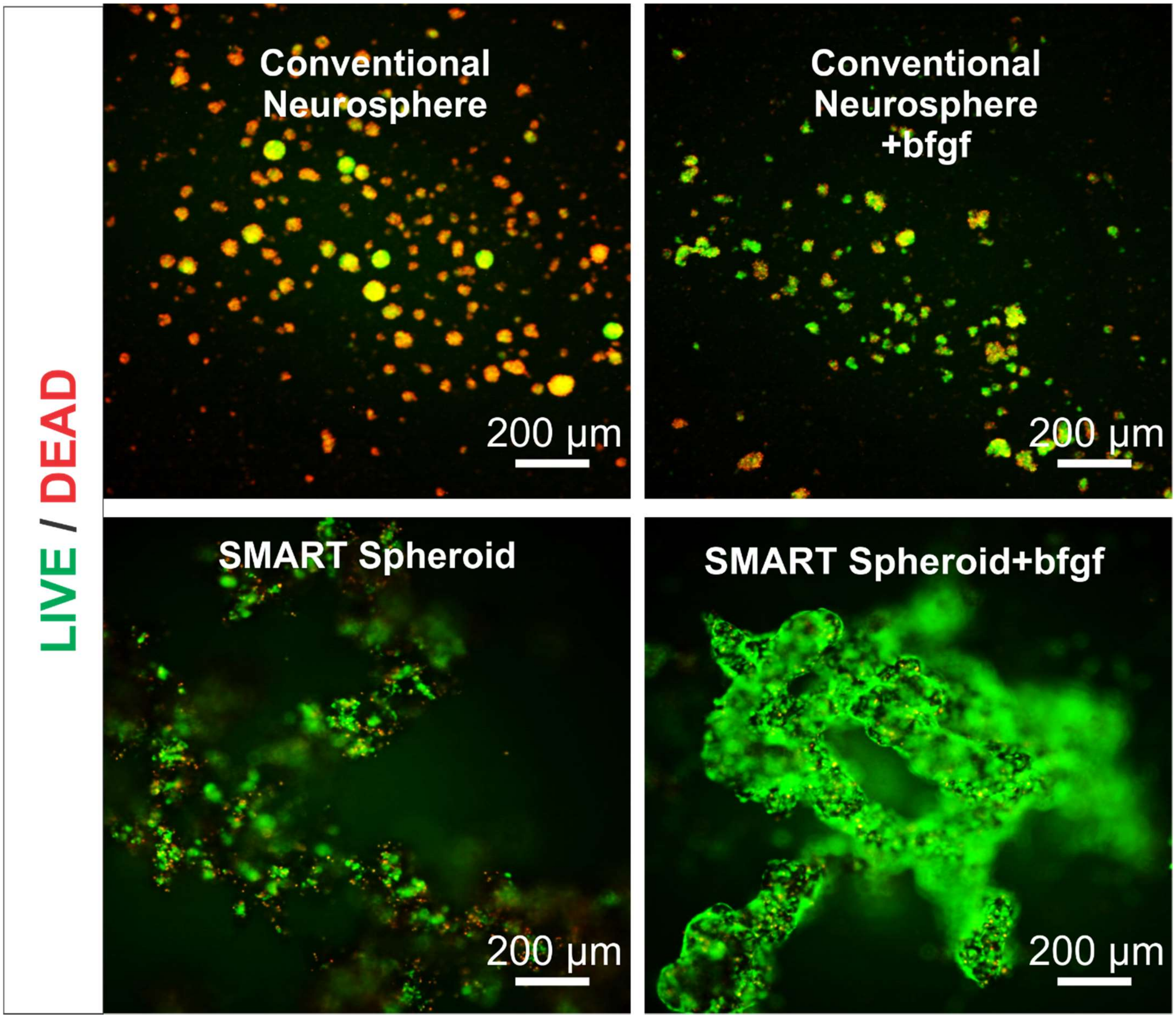

SUPPORTING FIGURE 8: *In vitro* inflammatory survival assay. Control and SMART Spheroids, either with or without bFGF, were exposed to conditioned media from activated macrophages. LIVE/DEAD stain (live-green, dead-red) was used to visualize cells, showing the robust survival of cells in the bFGF loaded SMART Spheroid condition.

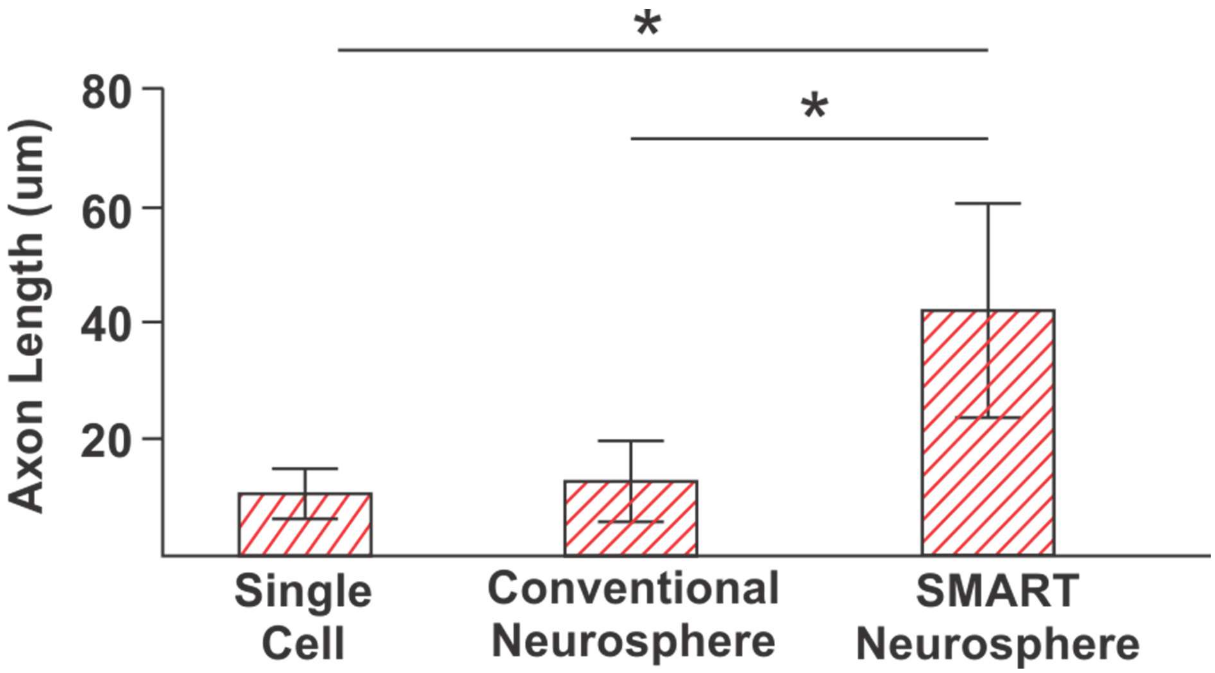

SUPPORTING FIGURE 9: Analysis of axon lengths of transplanted cells that differentiated into neurons (GFP+ and Tuj1+). Data are mean ± s.d. analysis of at least 50 cells per condition across n=3 animals, * $P < 0.05$ by one-way ANOVA.

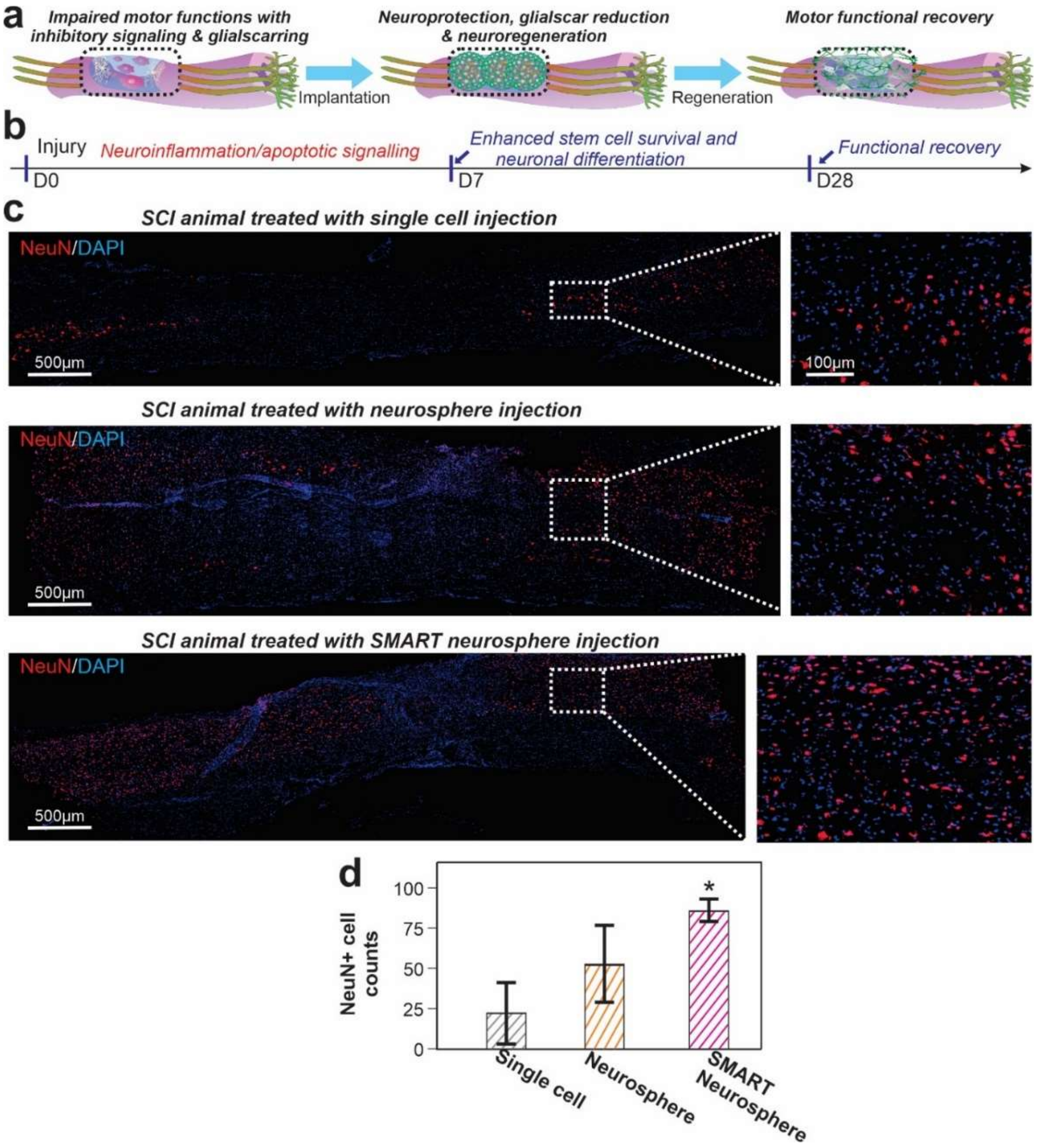

SUPPORTING FIGURE 10: Neuroprotective effect of SMART spheroids. (a-b) Analysis of NeuN+ cells in the spinal cord after injury and treatment with SMART neurospheres showing the neuroprotective effect of enhancing stem cell therapy. (c) Representative images of spinal cord sections stained for NeuN (red) motor neurons in the ventral horn, and DAPI (blue) cell nuclei. (d) Quantification of enhanced NeuN+ cell survival using ImageJ 1 month after treatment. Data are ± s.d. n = 1, * $P < 0.05$ by one-way ANOVA. Data points with the highest variance were removed equally across all groups.

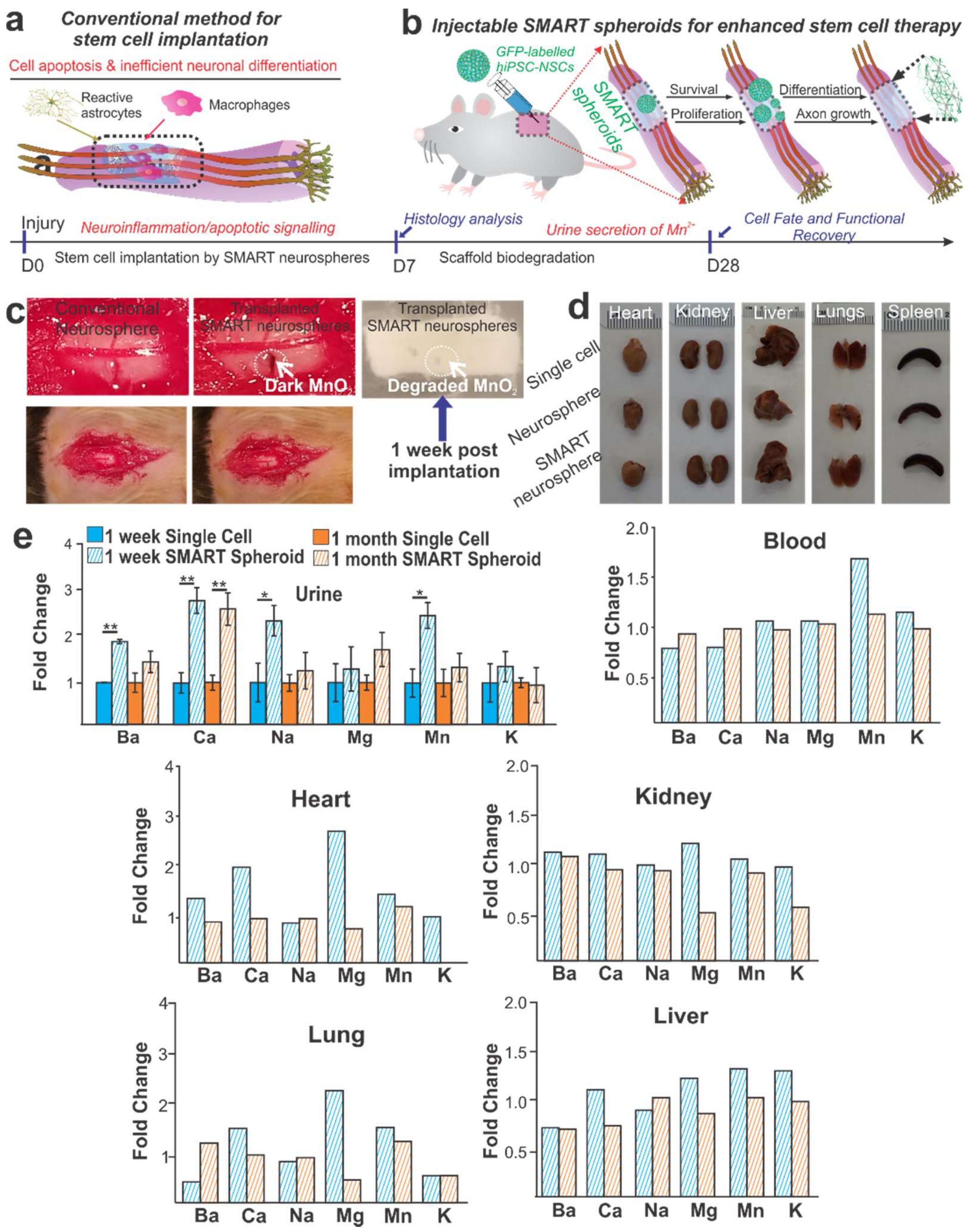

SUPPORTING FIGURE 11: Biocompatibility of SMART Spheroids *in vivo.* (a-b) Schematic diagram illustrating the timeline for in vivo scaffold implantation assay. (c) Representative photographs showing the implantation of cells and SMART neurospheres (dark-colored). (d) *In vivo* organ harvesting from animals after sacrificing at 1-week post-surgery and implantation. No significant differences were observed in the size or morphology of organs in the SMART Spheroid (S-NS) condition compared to neurosphere (NS) or single-cell (SC) controls. (e) Ion analysis in urine, blood, and other organs for Mn^{2+} ions and various other common ionic species was analyzed using ICP-MS. All graphs show data for short-term and long-term SMART neurosphere conditions normalized to single-cell treated conditions. As shown, organ-specific manganese ion concentration analysis shows little accumulation of degradation products of the scaffold (Mn) in common organs that show toxicity during Mn compound treatments. For data with statistical information data shown is mean ± s.d. n = 3, * $P < 0.05$ ** $P < 0.01$

GENE	Forward Primer	Reverse Primer
GAPDH	5'-CCGCATCTTCTTTTGCGTCG-3'	5'-GCCCAATACGACCAAATCCGT-3'
FAK	5'-GCTTACCTTGACCCCAACTTG-3'	5'-ACGTTCCATACCAGTACCCAG-3'
NOTCH2	5'-CCTTCCACTGTGAGTGTCTGA-3'	5'-AGGTAGCATCATTCTGGCAGG-3'
TuJ1	5'-GGTGTCCGAGTACCAGCAGT-3'	5'-TTCGTACATCTCGCCCTCTT-3'
GFAP	5'-CTGCGGCTCGATCAACTCA-3'	5'-TCCAGCGACTCAATCTTCCTC-3'

SUPPORTING TABLE 1: Primers used for quantitative PCR gene analysis. All primers were obtained from the PrimerBank database.

Mouse #	Week	Treatment	Slide #	Liver	Kidney
2	1	SMART	1	NNF	NNF
3	1	SMART	2	NNF	NNF
4	1	SMART	3	NNF	NNF
5	1	Control	4	NNF*	NNF
6	1	Control	5	NNF	NNF
7	1	Control	6	NNF	NNF
5	4	SMART	7	NNF	NNF
6	4	SMART	8	NNF	NNF
8	4	SMART	9	NNF	NNF
1	4	Control	10	Multifocal hepatocellular degeneration and necrosis with mixed inflammation	NNF
3	4	Control	11	NNF	NNF
11	4	Control	12	NNF	NNF
Notes: *Incidental finding. Liver mononuclear cell accumulation.					
Notes: Because multifocal necrotic foci were in a control mouse, the finding is presumed background and not treatment-related.					

SUPPORTING TABLE 2: Analysis of histological tissue where no notable findings (NNF) were found in any of the SMART neurosphere conditions in the short term (1 week) or long term (4 weeks) in the liver or kidney tissues, which are common organs for processing toxins in the body.

Supporting Movie 1. Representative animal behavior analysis for BMS scoring
